# Broad Epitope Coverage of Therapeutic Multi-Antibody Combinations Targeting SARS-CoV-2 Boosts In Vivo Protection and Neutralization Potency to Corner an Immune-Evading Virus

**DOI:** 10.3390/biomedicines12030642

**Published:** 2024-03-13

**Authors:** Ilse Roodink, Maartje van Erp, Andra Li, Sheila Potter, Sander M. J. van Duijnhoven, Milou Smits, Arthur J. Kuipers, Bert Kazemier, Bob Berkeveld, Ellen van Geffen, Britte S. de Vries, Danielle Rijbroek, Bianca Boers, Sanne Meurs, Wieger Hemrika, Alexandra Thom, Barry N. Duplantis, Roland A. Romijn, Jeremy S. Houser, Jennifer L. Bath, Yasmina N. Abdiche

**Affiliations:** ImmunoPrecise Antibodies Ltd., 4837 Amber Valley Parkway, Suite 11, Fargo, ND 58104, USA; mvanerp@ipatherapeutics.com (M.v.E.); ali@ipatherapeutics.com (A.L.); spotter@ipatherapeutics.com (S.P.); svanduijnhoven@ipatherapeutics.com (S.M.J.v.D.); msmits@ipatherapeutics.com (M.S.); akuipers@ipatherapeutics.com (A.J.K.); bkazemier@ipatherapeutics.com (B.K.); bberkeveld@ipatherapeutics.com (B.B.); evangeffen@ipatherapeutics.com (E.v.G.); bdevries@ipatherapeutics.com (B.S.d.V.); drijbroek@ipatherapeutics.com (D.R.); bboers@ipatherapeutics.com (B.B.); smeurs@ipatherapeutics.com (S.M.); whemrika@ipatherapeutics.com (W.H.); athom@ipatherapeutics.com (A.T.); barryduplantis@hotmail.com (B.N.D.); rromijn@ipatherapeutics.com (R.A.R.); jeremyhouserphd@gmail.com (J.S.H.); jbath@ipatherapeutics.com (J.L.B.);

**Keywords:** antibody-dependent cellular effects, bio-engineering, COVID-19, multi-antibody combination, neutralizing antibodies, resilient efficacy, SARS-CoV-2

## Abstract

Therapeutic antibodies (Abs) which act on a broader range of epitopes may provide more durable protection against the genetic drift of a target, typical of viruses or tumors. When these Abs exist concurrently on the targeted antigen, several mechanisms of action (MoAs) can be engaged, boosting therapeutic potency. This study selected combinations of four and five Abs with non- or partially overlapping epitopes to the SARS-CoV-2 spike glycoprotein, on or outside the crucial receptor binding domain (RBD), to offer resilience to emerging variants and trigger multiple MoAs. The combinations were derived from a pool of unique-sequence scFv Ab fragments retrieved from two SARS-CoV-2-naïve human phage display libraries. Following recombinant expression to full-length human IgG_1_ candidates, a biolayer interferometric analysis mapped epitopes to bins and confirmed that up to four Abs from across the bins can exist simultaneously on the spike glycoprotein trimer. Not all the bins of Abs interfered with the spike protein binding to angiotensin converting enzyme 2 (ACE2) in competitive binding assays, nor neutralized the pseudovirus or authentic virus in vitro, but when combined in vivo, their inclusion resulted in a much stronger viral clearance in the lungs of intranasally challenged hamsters, compared to that of those treated with mono ACE2 blockers. In addition, the Ab mixtures activated in vitro reporter cells expressing Fc-gamma receptors (FcγRs) involved in antibody-dependent cellular cytotoxicity (ADCC) and phagocytosis (ADCP). The best four-Ab combination neutralized seventeen variants of concern from Wuhan-Hu1 to Omicron BA.4/BA.5 in vitro.

## 1. Introduction

The characteristic surface glycoprotein (spike protein or S-protein) of SARS-CoV-2 is the mediator of host–cell attachment and the entry of the virus [1] and is thus indispensable for the infection causing COVID-19 in humans. Therefore, the disruption of the interaction of the receptor-binding domain (RBD) of this protein with the host target, angiotensin converting enzyme 2 (ACE2), is a sensible COVID-19 intervention. For this reason, most SARS-CoV-2-directed monoclonal antibodies (Abs), which are intended to provide acute therapeutic protection from the disease after administration, target epitopes clustered at or close to the ACE2-binding interface of the RBD region [2].

Vaccination, which also relies on the spike protein as an antigen, provides substantial protection against severe disease but is less effective in immunocompromised individuals, those with certain underlying health conditions such as heart or lung disease or cancer, and the elderly. These individuals are also the most at risk for progression to severe disease upon infection, requiring urgent interventions with therapeutic agents such as monoclonal antibodies. Effective early intervention in all (vaccinated) patients may also help mitigate the risk of post-acute sequelae of COVID-19 (PASC) or ‘long-haul COVID-19′ which may last over a year post-infection and place a substantial burden on recovering patients [3]. Furthermore, the continued emergence of new viral variants may impact vaccine efficacy. In that respect, it is noteworthy that Omicron sublineages, including CA.3.1, CH.1.1, and XBB.1.5, nearly completely escape neutralizing antibodies induced by three doses of mRNA vaccination, with CA.3.1 and CH.1.1 being highly resistant to bivalent mRNA vaccination as well [4]. This might increase the risk of emerging breakthrough infections, undermine controlling the virus by vaccination alone, and necessitate the presence of effective therapies such as monoclonals as treatment options.

However, targeting a crucial element of a virus’s life cycle puts selective pressure on it to escape from acquired immunity by accumulating mutations within the RBD [5,6]. Therefore, combination therapies were quickly developed [7,8,9] which were efficacious in real life [10,11,12]. However, due to immune evasion as a consequence of the strong selective pressure on the RBD, therapies that rely on one or two antibodies targeting the RBD region alone lost efficacy against emerging variants of concern (VoCs), in particular, Omicron. Several antibody therapies, including bamlanivimab + etesevimab, bebtelovimab, cilgavimab + tixagevimab, regdanvimab, casirivimab + imdevimab, and sotrovimab, that acquired emergency use approval by the United States Food and Drug Administration (FDA) were subsequently revoked because they all became ineffective against Omicron variants [13,14,15]. Currently, there are no FDA-approved antibody therapies for Omicron.

The SARS-CoV-2 virus will endure by the genetic evolution of currently circulating variants into new lineages to increase its odds to escape acquired immunity and/or to transmit faster and easier among the human population [16,17,18]. As COVID-19 is life-threatening for large groups of vulnerable people, powerful therapies resilient to a persistent and ever-changing virus are urgently needed. Increasing the number of preferably conserved, distinct epitopes located on multiple domains and functional structural elements, not exclusively residing on the RBD, and targeting these simultaneously using a mixture of Abs is an encouraging strategy [19,20,21]. Therapies comprising more than two combinatorial Abs targeting diversified distinct epitopes are uncommon. The three Abs in ZMapp^TM^ are successful in the treatment of Ebola viral infections [22]. In another example, a six-membered Ab combination, G03-52-01, to botulinum neurotoxin serotypes A and B prevented botulism in guinea pigs [23]. As for SARS-CoV-2 treatments, a combination of three human and humanized anti-RBD Abs, possibly supplemented with an anti-N-terminal domain (NTD), has been suggested [24]. In IMM-BCP-01, which is a patient-derived three-Ab combination, two Abs block ACE2 binding, and the third Ab alters the conformation of the S-protein trimer [9]. This combination is described to be active across Wuhan-Hu1 to Omicron BA.1 and BA.2 VoCs.

Besides enhanced resilience against the evolution of immune evasion, the concurrent binding of multiple Abs may induce higher-order anti-viral activities. When these mechanisms work in concert, they are anticipated to intensify the viral-load-reducing effect. The aim of this study was to discover and engineer a virus-neutralizing antibody combination consisting of synergistically interacting RBD-blocking and non-RBD-blocking Abs aiming for broad epitope coverage across the full-length spike trimer, thereby purposely including antibodies targeting more conserved epitopes present in the S2 subunit and C-terminal domain of the S1 subunit. To this end, a diversity-focused discovery approach was applied followed by an extensive characterization of the effect of higher-order combinations on simultaneous binding and neutralization. This approach is a clear discriminator compared to the design of the combination therapies on the market thus far. Our multi-antibody combination should offer a sustainable and potent therapy for the prevention or treatment of COVID-19 caused by the ever-evolving SARS-CoV-2 virus.

## 2. Materials and Methods

### 2.1. Materials

#### 2.1.1. Synthetic Genes

Synthetic genes encoding for SARS-CoV-2 S-protein variants, including B.1.1.7 (Alpha), B.1.351 (Beta), B.1.617.2 (Delta), P.1 (Gamma), B.1.429 (Epsilon), B.1.526 (Iota), B.1.621 (Mu), and C.37 (Lamda) variants; Omicron B.1.1.529.1 (BA.1) and B.1.1.529.2 (BA.2) sublineages; and the A (Wuhan-Hu1) and B (D614G) parental lineages, were obtained from GeneArt (Thermo Fisher Scientific, Regenburg, Germany).

#### 2.1.2. Reagents

HRP-conjugated rabbit anti-human IgG (H + L) (cat.# 6140-05), and goat anti-human IgG (cat.# 2040-05) detection reagents were purchased from SouthernBiotech (Birmingham, AL, USA). HRP-conjugated mouse anti-M13 antibody (cat#11973-MM05T-H) was purchased from Sino Biological (Beijing, China) and HRP-conjugated rabbit anti-mouse IgG from Dako (Agilent, Santa Clara, CA, USA, cat# P0260). Mouse anti-VSV tag, clone P5D4 was produced by IPA (Oss, Netherlands, cat# MQ20.101-100). Donkey anti-human IgG antibodies conjugated to phycoerythrin (PE) were bought from Abcam (cat.# ab102439). BSA (cat# A3912), Tween-20 (cat# P1379), H_2_SO_4_ (cat# 339741), and ethanolamine (cat# E0135) were bought from Sigma-Aldrich (Merck KgA, Darmstadt, Germany). TMB (cat# 10354603) was bought from Thermo Fisher Scientific, Regenburg, Germany), and formaldehyde 4% (cat# 11699404) from VWR International (Randor, PA, USA)

Human ACE2-hFc, SARS-CoV-1 spike trimer, RBD, and RBD-hFc were produced using the rPEx^®^ recombinant protein production platform using HEK293 cells (ImmunoPrecise Antibodies (IPA), Utrecht, The Netherlands,). Wild-type SARS-CoV-2 (Wuhan-Hu1) S-protein trimer was obtained from the Canadian National Research Council (cat.# A33-11-02-SMT1) or purchased from Cube Biotech (Monheim, Germany, cat.# 28702). Various spike protein subunits, including AVI-tagged NTD-hFc, S1-His, S1-hFc, S1-mFc, S2-ECD-hFc, and S1-S2-His, were purchased from Sino Biological (Beijing, China, cat.#’s 40591-V41H, 40591-V08H, 40591-V02H, 40591-V05H1, 40590-V02H, and 40589-V08B1, respectively). Spike protein trimers reflecting B.1.1.7, B.1.351, B.1.429, B.1.525, B.1.617, B.1.617.2 (Delta), and P.1 were obtained from Cube Biotech (Monheim, Germany cat.#’s 28717, 28720, 28737, 28733, 28740, 28745, and 28723, respectively).

Benchmark antibodies B38 (PDB ID: 7BZ5) [25], casirivimab (REGN10933) [26], etesevimab (CB6) [27], CR3022 [28], H4 [25], and imdevimab (REGN10987) [26] and control antibodies bococizumab [29], CNTO607 [30], and trastuzumab [31,32] were generated from published sequences and expressed as human IgG_1_ in HEK293 cells via the rPEx^®^ platform (ImmunoPrecise Antibodies (IPA), Utrecht, The Netherlands). This platform was also used to produce benchmark VHH72 [33], SB14 [34], and SB68 [35] as VHH-hFc1 molecules and to produce candidate anti-S-protein antibodies as human IgG_1_ or IgG_1_-Fab. For the functional testing, prioritized anti-spike antibodies were produced in CHO cells. Human IgG_1_ and Fab molecules were purified using MabSelect SuRe LX (Cytiva, Danaher, Washington, DC, USA, cat #17-5474-02) or CaptureSelect CH1-XL (ThermoFisher Scientific, Waltham, MA, USA, cat #29-3219-05 ) affinity purification, respectively. The binders were polished by size-exclusion chromatography using a Superdex200 Increase column (Cytiva, Danaher, Washington, DC, USA, cat #29-3219-05) that was equilibrated in PBS, sterilized, and stored at either at 4 °C (short term) or −80 °C (long term).

### 2.2. Phage Display Library Panning

Two phage display libraries from healthy (#0899, IPA) and auto-immune-diseased (#0845, IPA) SARS-CoV-2-unexposed donors with a complexity of 1 × 10^10^ and 1 × 10^11^ colony-forming units, respectively, were rescued using *Escherichia coli* TG1 cells (Immunosource, Schilde, Belgium, cat# 60502-2) and helper phages (Immunoprecise Antibodies, Oss, the Netherlands) and interrogated for antibody fragments binding to the spike protein reflecting the Wuhan-Hu1 strain using standard methods [36]. For target enrichment of phage particles, eleven panning strategies were tested in parallel using varying combinations of S1 + S2 and RBD reflecting various SARS-CoV-2-associated spike protein (fragments). Depletion panning with SARS-CoV-1 spike trimer protein, benchmark antibody CR3022 [28], or human ACE2 bound to the S-protein or with irrelevant non-target proteins led to the discovery of binders to specific epitopes displaying no or limited off-target reactivity. Approximately 700 clones were selected for monoclonal scFv expression. Upon confirming their target specificity by ELISA, the top 279 periplasmic fractions were selected for initial functional assessment resulting in 60 unique-sequence scFv clones.

### 2.3. ELISA Screening

Per well, 50 µL of 1.5 µg/mL target in 50 mM carbonate at pH of 9.6 was incubated overnight at 4 °C in Bio-One High Bind ELISA plates (Greiner, Kremsmünster, Austria). Unused reactive sites were blocked with 1% (*m*/*v*) BSA in PBS for 60 min. If required, an additional capture step was conducted in PBS for 1 h at room temperature (RT). Coated plates were washed with PBS enriched with 0.05% (*v*/*v*) Tween-20 (PBS-T). Serial dilutions of phage or recombinant Abs in PBS supplemented with 5% (*m*/*v*) skim milk or 1% (*m*/*v*) BSA were applied in duplicate and incubated for 60 min at RT. After washing with PBS-T, an appropriate HRP-conjugated secondary antibody (anti-M13 or goat anti-human IgG) was added and incubated for 60 min at RT. Target-bound scFvs were detected by incubation with mouse anti-VSV followed by anti-mouse IgG-HRP. After final washing, wells were typically stained with 50 µL TMB substrate for 10 min and quenched by the addition of 50 µL 2 M H_2_SO_4_. Absorbance was read at λ 450 nm on an Envision multimode plate reader(PerkinElmer, Waltham, USA), and data were processed using GraphPad Prism (La Jolla, CA, USA, version 9).

### 2.4. Biolayer Interferometric Interaction Analysis

All label-free interaction analyses were performed on an Octet HTX biolayer interferometry-based detection system (Sartorius, Göttingen, Germany) operated with either AR (amine-reactive), SAX (streptavidin-coated), or AHC (anti-human-Fc capture) sensors. Experiments were conducted at 25 °C using PBS fortified with 0.05% (*v*/*v*) Tween-20 and 0.1% (*m*/*v*) BSA.

Combinatorial pairwise Ab competition or “epitope binning” assays were performed as follows. In a “classical sandwich” assay format, Abs were covalently coupled onto AR sensors using standard coupling conditions and used to capture S1(D614G)-His monovalent target (typically 5 µg/mL at 65 nM) followed by an Ab analyte (typically at 10 µg/mL). Alternatively, reaction surfaces were generated by coating SAX sensors with 5 µg/mL biotinylated Abs. Surface-immobilized Abs were regenerated with 75 mM phosphoric acid.

“Waterfall” experiments were conducted on freshly ACE2-coated SAX sensors (single use, not regenerated) using 5 µg/mL S1-His(D614G) followed by an Ab titration spanning 6.0 μM to 25 nM binding sites as a six-membered three-fold dilution series, with one concentration (667 nM) in duplicate.

“Tandem cocktail” multi-Ab binning experiments were performed on SAX sensors coated with 5 µg/mL biotinylated Ab 23-H7 and used to tether 5 µg/mL S-protein trimer. Three Ab analytes from non-overlapping bins were associated in consecutive analyte binding steps at 60 µg/mL. Each step included the Abs from the previous steps, thereby maintaining the levels of each Ab analyte applied for the duration of the assay.

Alternatively, multi-Ab binnings were performed in a “premix” assay format using SAX sensors coated with 5 µg/mL biotinylated Abs from different bins (2, 4, C, or S2), or controls, including biotinylated ACE2-hFc or mouse anti-His mAb. Spike protein trimer (0.5 µM binding sites) was premixed with Abs, either individually or as two-, three-, or four-membered combinations at saturating concentrations (10 µM to 30 µM binding sites). Samples of premixed S-protein/Ab complexes, S-protein alone, or buffer were used as analytes for binding to the Ab-coated sensor (or control surfaces) to probe for free binding sites in these mixtures. Binding responses were compared with those of S-protein alone and determined to be blocked if their responses were significantly suppressed to baseline levels comparable to that of the blank buffer.

Data were processed in Sartorius Octet Data Acquisition software v.12.0.1.55 by Y-aligning to zero at each association step and further analyzed in the Octet Data Analysis HT v.12.0.1.8, Epitope Binning module (Sartorius, Göttingen, Germany). Heat maps were curated manually in Microsoft Excel by merging the results from different experiments.

### 2.5. Cell-Associated S-Protein Screening

Using the FectoPRO system (cat#116-100) as specified by the manufacturer (PolyPlus Transfection, Illkirch, France)), HEK293F cells were transiently transfected with a mammalian expression vector (IPA Europe) into which synthetic genes encoding S-protein variants were cloned. Cells expressing S-protein trimers were harvested 48 h post-transfection, washed, and dispensed to 384-well plates with 1.0 × 10^5^ cells per well. Serial dilutions of test or control Abs were added in a final volume of 30 µL per well in triplicate. After 1 h, the wells were washed, and Ab binding was detected with donkey anti-human IgG-PE. Following fixation using 2% (*v*/*v*) formaldehyde, cells were analyzed using an iQue Screener high-throughput flow cytometer (Sartorius).

### 2.6. Pseudovirus Neutralization (Utrecht University)

The top periplasmic fractions, selected after the panning of pre-existing human scFv repertoires and confirmation of their target-specificity by ELISA, were nominated for initial functional assessment using a pseudovirus neutralization assay described here. Initial functional validation of recombinant versions of clones selected for further analysis was performed with this assay as well.

The production of VSV virus particles expressing the SARS-CoV-2 S-protein is described elsewhere [37]. Briefly, S-protein genes cloned into the pCAGGS expression vector system were transfected into HEK293T cells. Cells were then infected with the VSV∆G pseudotyped virus and further modified to encode the *Photinus pyralis* luciferase reporter protein. After 24 h, supernatants were collected and titrated on African green monkey VeroE6 cells expressing full-length human transmembrane serine protease 2 (TMPRSS2). Test Abs were diluted in DMEM cell-growth medium supplemented with 1% (*v*/*v*) fetal calf serum (Bodinco, Alkmaar, the Netherlnds), 100 U/mL penicillin, and 100 µg/mL streptomycin before being added to pseudotyped virus particles and incubated for 1 h at RT. The mixture was added to a confluent monolayer of VeroE6 cells in a 96-well tissue culture plate and incubated for 24 h. Following washing and lysis of the cells, luciferase activity was measured in the presence of D-luciferin substrate (Promega, Madison, WI, USA) using a Centro LB960 plate luminometer (Berthold, Bad Wildbad, Germany). Neutralization was calculated as the ratio of luciferase activity in the presence of Abs normalized to a negative control well containing only pseudotyped virus and assay buffer.

### 2.7. Pseudovirus Neutralization (NIAID/NIH)

Except for the initial screening of periplasmic fractions and first functional validation of recombinant versions of selected clones, all other pseudovirus neutralization screenings were carried out using the method described here. The protocol and validation of this assay have been reported elsewhere [38]. Briefly, pseudovirus particles were produced using a backbone vector (F-lucP.CNDO∆U3) encoding the HIV genome, and the full-length S-protein genes were cloned into pCXAS-PXMX. Vectors were transfected into HEK293 cells for pseudovirus particle production harvested on day 2 post-transfection. To test neutralization, pseudovirus was pre-incubated with Abs in a serial dilution series at a starting concentration of 100 µg/mL for 1 h at 37 °C, and the complexes were added to HEK293 cells, which had been transfected the previous day with human ACE2 and TMPRSS2 coding sequences. On day 3 of the assay, Steady Glo (Promega, Madison, WI, USA) was added to each well and reactions were quantified using a luminometer.

### 2.8. Authentic Virus Neutralization

Authentic virus neutralization assays were conducted by Prof. Dr. Oliver Keppler and co-workers (Ludwig-Maximilians University, Munich, Germany) using a method adapted from Wratil et al. [39]. Briefly, virus stocks (prototypic variant strains EU-1 (D614G), Alpha, Beta, Delta, and Omicron BA.1, BA.1.1, BA.2, and BA.4/BA.5) were produced by infection of VeroE6 cells (American Type Culture Collection, ATCC) grown in virus expansion medium (DMEM containing 5% fetal calf serum, 100 U/mL penicillin–streptomycin). Virus stocks were titrated for TCID_90_ using MDA-MB-231 cells (ATCC) overexpressing human ACE2. For infection neutralization screening, cells were cultured and infected in 384-well plates (10,000 cells per well). The respective TCID_90_ of each virus stock was incubated for 1 h with dilution series of each test compound at a starting concentration of 50 µg/mL. Subsequently, 10 µL of the virus–compound mixtures were added to 20 µL expansion medium and added to wells containing MDA-MB-231-hACE2 cells. At 48 h post-infection, cytopathic effects were determined by the addition of 10 µL CellTiter-Glo 2.0 (Promega, Madison, WI, USA) and subsequent measurement of bioluminescence signals (0.5 s integration time, no filter) to quantify virus-mediated cytotoxicity in target cells.

### 2.9. ADCC/ADCP Reporter Assays

In the case of ADCC analyses, SARS-CoV-2-S CHO-K1 (Promega, Madison, WI, USA) target cells were either incubated with a four-fold dilution series from 15 μg/mL to 230 pg/mL test Ab or with a semi-^10^log dilution series from 31.6 μg/mL to 3.16 ng/mL test Ab, in the presence of Jurkat ADCC reporter cells (Promega, Madison, WI, USA) at a cell ratio of 4:1.

Similarly, ADCP analyses were performed using THP-1 reporter cells (Promega, Madison, WI, USA) at a cell ratio of 3:2 to SARS-CoV-2-S CHO-K1 target cells incubated with a semi-log dilution series of 10 μg/mL to 150 pg/mL Ab.

The Ab combinations and control NISTmAb were assayed in triplicate. In each run, samples without the addition of SARS-CoV-2-S CHO-K1 target cells were taken along to monitor any non-specific effects. After incubation for 4 h (ADCP) or 6 h (ADCC) at 37 °C, Bio-Glo (ADCC) or Bio-Glo NL (ADCP) substrate (Promega, Madison, WI, USA) was added to the antibody–cell mixtures. Luminescence was measured after 5 to 10 min with an Envision spectrophotometer. Each ADCC and ADCP run was conducted in triplicate.

### 2.10. In Silico Developability Analysis and Manufacturability Optimization

Three variable fragment (Fv) homology models per Ab candidate were generated using Schrödinger BioLuminate (Schrödinger, Mannheim, Germany, version 2021-01) and subsequently analyzed for AggScore, pI, hydrophobic moment, and HCDR3 length [40]. The Fv sequences of trastuzumab and bococizumab were taken along as clinically evaluated references.

Using the same software, lead antibodies were analyzed for surface-based reactive residues following sequence-based alignment. The amino acids surrounding the putative N-glycosylation site were analyzed for germline homology. Due to high local homology, Asn (N) was replaced with the germline Lys (K) for 22-D9. In the case of clone 21-F2, the most closely related amino acid, Gln (Q), was used. See Figure S1 for an example of a homology model.

### 2.11. HPLC-Based Analyses

All measurements were performed on a bio-inert Agilent 1260 Infinity II HPLC (Agilent, Santa Clara, CA, USA) system equipped with an autosampler, binary pump, and diode-array detector. Data were recorded at λ 215 nm and analyzed using OpenLab Chemstation software (Agilent, Santa Clara, CA, USA, version 3.5.2.275). See Figure S1 for an example of HPLC-based assays.

#### 2.11.1. Size-Exclusion Chromatography (SEC)

An amount of 5 μg of sample (5 μL of 1 mg/mL) was analyzed on an Agilent AdvanceBio SEC column (2.7 µm, 300 Å, 7.8 × 300 mm) (Agilent, Santa Clara, CA, USA) using 50 mM sodium phosphate, 0.4 M NaClO_4_ at pH of 7.0 as mobile phase for 20 min and at a flow rate of 1.0 mL/min.

#### 2.11.2. Cross-Interaction Chromatography (CIC)

With modifications, CIC was carried out according to Sun et al. [41]. In brief, a CIC column was prepared by coupling approximately 30 mg of human polyclonal IgG (Sigma-Aldrich, Merck KgA, Darmstadt, Germany), cat#I4506) on a HiTrap NHS-activated resin (Cytiva Danaher, Washington, DC, USA, cat#17-0716-01) followed by quenching with ethanolamine. Analyses were performed by injection of 5 μg of sample (5 μL out of 1 mg/mL) using 50 mM sodium phosphate and with 0.4 M NaClO_4_ at pH of 7.0 as the running buffer. The run time was 30 min at a flow rate of 0.1 mL/min.

#### 2.11.3. Standup Monolayer Chromatography (SMAC)

SMAC analyses were performed using a method adapted from Kohli et al. [42]. Briefly, 5 μg of sample (5 μL of 1 mg/mL) was injected into an Agilent SEC-3 column (300 Å, 4.6 × 300 mm) (Agilent, Santa Clara, CA, USA) using a running buffer at pH of 7.0 containing 50 mM sodium phosphate and 0.4 M NaClO_4_ for 30 min at a flow rate of 1.0 mL/min. Recombinant humanized IgG_1_κ reference NISTmAb and trastuzumab with biopharmaceutical grade properties, as well as bococizumab [29] and CNTO607 [30] benchmark antibodies with reported poor biophysical properties were used as reference.

### 2.12. Capillary Electrophoresis (CE)

Capillary electrophoresis was conducted on a LabChip GXII Touch HT (PerkinElmer, Waltham, USA) under reducing or non-reducing conditions at 1 mg/mL protein according to the instructions of the instrument producer. Data were processed using LabChip GX Reviewer (version 5.5.2312.0). See Figure S1 for an example of assay.

### 2.13. Assessment of Solubility

Control (NISTmAb, CNTO607) or candidate Abs at 1.0 mg/mL in PBS were incubated with PEG4000 to obtain dose–response data (2.7% to 25% (*m*/*v*) PEG4000 Sigma-Aldrich, Merck Kga, Darmstadt, Germany, cat# 9727) prepared through 1.25-fold dilutions in eleven steps) for 2 h at RT with an orbital plate shaker. The turbidity of samples was measured spectrophotometrically at λ 350 nm. See Figure S1 for an example of assay.

### 2.14. In Vivo SARS-CoV-2 Challenge of Hamsters

**Figure 1:**
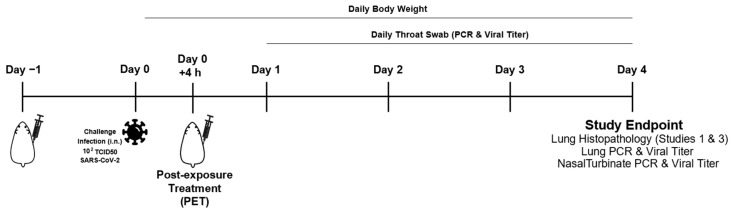
Study design for the SARS-CoV-2 infection challenge of Syrian golden hamsters and study measures.

Animal studies were conducted at ViroClinics Xplore (Schaijk, The Netherlands) as previously described [43,44] in compliance with European Union Directive 2010/63/EU and the standards of Dutch law for animal experimentation. Ethical approval was registered under project license numbers AVD277002015283-WP26 and -WP33. Briefly, specific pathogen-free male Syrian golden hamsters (*Mesocricetus auratus*) aged 9 to 10 weeks at the start of the experiment were randomly assigned to experimental cohorts of five animals each. The hamsters were housed in elongated Type 2 cages with one or two animals per cage under BSL-III conditions during the experiment. Antibody or mock (PBS) solutions were administered as a single intraperitoneal injection at indicated times (Figure 1). All animals were challenged on day 0 with a single intranasal administration of 10^2.0^ TCID_50_ SARS-CoV-2 (BetaCoV/Munich/BavPat1/2020) in a total dose volume of 100 µL administered equally in both nostrils. Animals were weighed daily, and their throats swabbed.

On day 4 post-challenge, all animals were euthanized by abdominal exsanguination under isoflurane anesthesia (3–5%). Throat swabs and right lung and nasal turbinate tissues were frozen for virological assessment by quantitative PCR and virus titration. In addition, samples of the left nasal turbinates, trachea, and entire left lung (often with the presence of the primary bronchi) were preserved in 10% (*v*/*v*) neutral-buffered formalin, embedded in paraffin, and micro-sectioned to 3 μm sections on glass slides. The sections were stained with hematoxylin and eosin for histopathological evaluation using an Olympus BX45 light microscope(Tokyo, Japan) with magnification steps of 40×, 100×, 200×, and 400× and subjected to blinded review by a third-party pathologist. The severity of inflammation was scored based on inflammatory cell infiltration in the tracheas and bronchi as follows: 0 = no inflammatory cells, 1 = few inflammatory cells, 2 = a moderate number of inflammatory cells, and 3 = many inflammatory cells.

### 2.15. Viral Load Quantification

For the determination of replication-competent virus levels, quadruplicate ten-fold serial dilutions were used to determine the virus titers in confluent layers of VeroE6 cells. In short, serial dilutions of throat swabs and tissue homogenates samples were prepared and incubated on VeroE6 monolayers for 1 h at 37 °C. The VeroE6 monolayers were washed and incubated for 5 or 6 days at 37 °C. Viability was scored using the vitality marker WST8. Viral titers, expressed as ^10^Log(TCID_50_/mL) or ^10^Log(TCID_50_/g), were calculated using the method of Spearman–Karber.

For detection, viral RNA was extracted from samples using Magnapure LC total nucleic acid isolation kit (Roche, Basel, Switzerland). RNA amplification and quantification were carried out on a 7500 RT-PCR System (Applied biosystems, Waltham, USA) using specific primers (E_Sarbeco_F: ACAGGTACGTTAATAGTTAATAGCGT and E_Sarbeco_R: ATATTGCAGCAGTACGCACACA) and probe (E_Sarbeco_P1: ACACTAGCCATCCTTACTGCGCTTCG) as described [45]. The number of RNA copies was calculated and denoted as ^10^Log(copies/mL) or ^10^Log(copies/g).

### 2.16. Statistical Analyses

The throat swab RT-PCR tests and throat swab virus titration of all hamster treatment groups were compared to those of the mock group. Mixed model analyses were conducted in SAS with Proc Mixed (SAS Institute, Cary, NC, USA). For the virology and histopathology variables measured on day 4 post-challenge, a two-sided *p*-value was calculated for Fisher’s exact test for categorical variables, and Wilcoxon rank-sum exact test was used for continuous and ordinal variables. Since the statistical analysis of these variables was explorative in nature, no correction for multiple testing was used. When appropriate, a Student’s *t* test was used to compare the results of two groups.

## 3. Results

### 3.1. Discovery and Engineering of an Antibody Panel with a Broad SARS-CoV-2 Spike Protein Epitope Footprint

**Figure 2:**
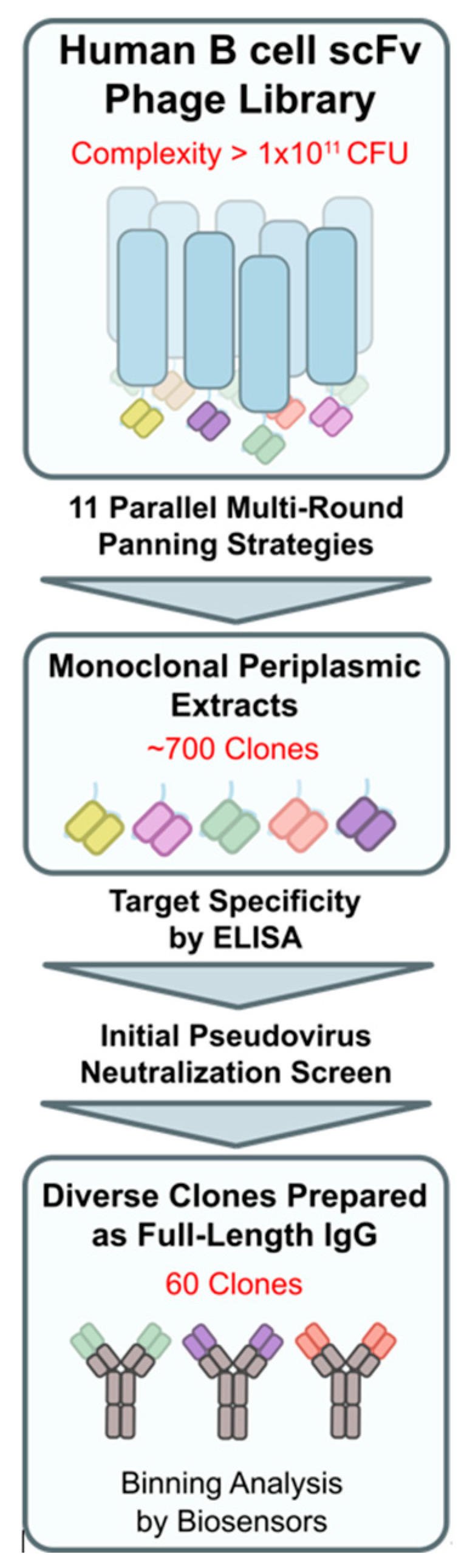
Discovery workflow overview for phage display panning up to candidate pool selection of 60 clones for binning analysis.

Eleven panning strategies, each with multiple (de-)selection rounds, were applied in parallel to interrogate two human, SARS-CoV-2-naïve phage display libraries. The discovery procedure yielded 60 unique-sequence and diverse-epitope Wuhan-Hu1 S-protein-binding scFv fragments (Figure 2). The down-selected fragments were recombined to full-length human IgG_1_ molecules and expressed in HEK293 and CHO cells. The reactivity of the purified immunoglobulins was confirmed by binding to plate-immobilized (ELISA) S-protein fragments, including streptavidin-labeled RBD (S1B), His-tagged S1, and S1 + S2-ECD. In addition, the blocking potency of promising and diverse-reactivity clones was assessed in a pseudovirus neutralization assay.

**Figure 3:**
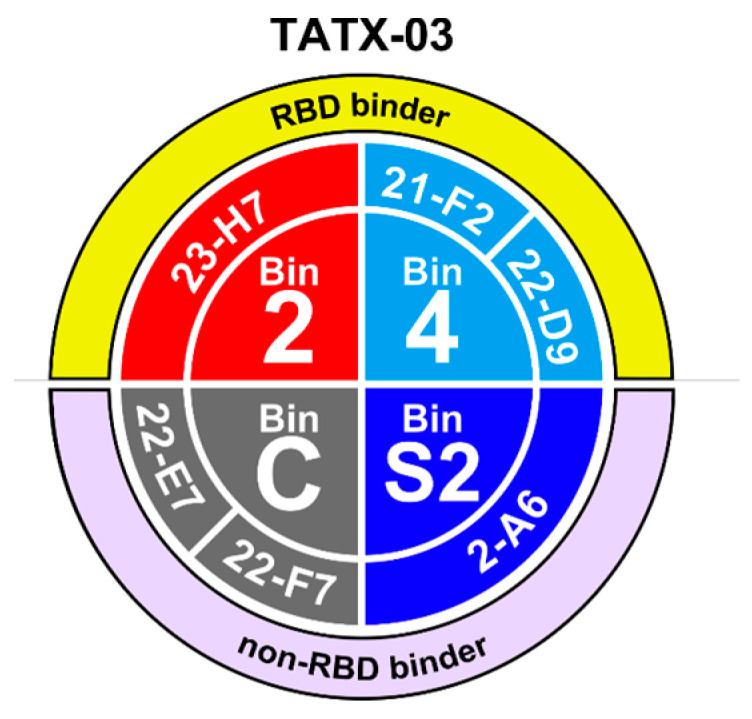
Bin and clone assignment of lead candidates, designated TATX-03. Bins 2 and 4 interfere with the interaction between RBD and ACE2, whereas bins S2 and C do not.

**Figure 4:**
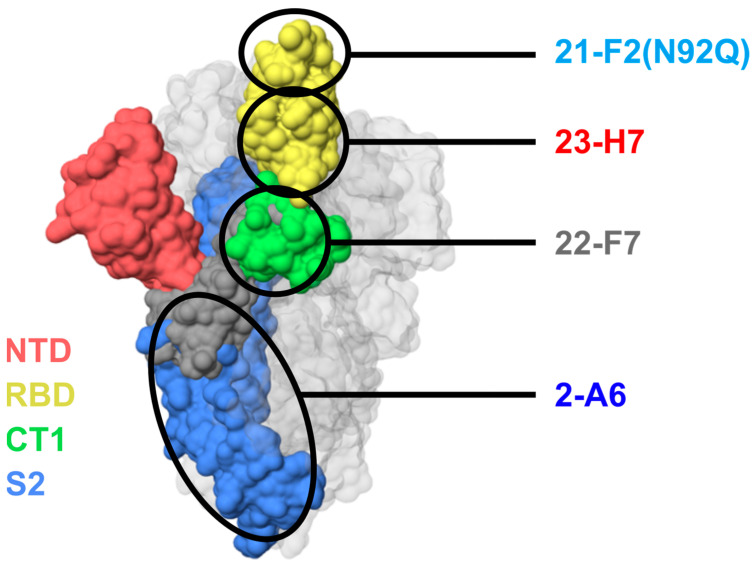
Predicted locations of epitope bins of TATX-03b′ clones 21-F2 (bin 4), 23-H7 (bin 2), 22-F7 (bin C), and 2-A6 (bin S2) based on relationships to reference clones and patterns of blocking. Colors indicate S-protein regions: NTD, N-terminal domain (red); RBD, receptor-binding domain (yellow); CT1, C-terminal domain S1 subunit (green); S2, S2 subunit (blue). Note that these colors do not correlate with the color scheme in Figure 3.

To meet the study’s need for a broad epitope coverage, an epitope map was created based on label-free biolayer interferometry-based screenings using various assay orientations, leading to the selection of a panel of six lead Abs spread across non-overlapping bins 2 (23-H7), 4 (21-F2 and 22-D9), C (22-E7 and 22-F7), and S2 (2-A6), collectively designated TATX-03 (Figure 3 and Figure 4).

For epitope mapping, the reactivity of Abs was first confirmed by determining their binding to immobilized S-protein fragments (RBD, S1, and S2). The Abs that bound RBD and S1 were further categorized by their blocking and non-blocking relationships while competing for binding to a monomeric S-protein subunit 1 (S1(D614G)-His), using a pairwise sandwich assay format. ACE2 and a panel of nine RBD-specific Abs with known epitopes and publicly available sequences were included and served as structural benchmarks that mapped the bins to the spike protein: imdevimab [26], casirivimab [26], etesevimab [27], B38 [25], H4 [25], CR3022 [28], VHH-72 [33], SB14 [34], and SB68 [35]. The results are summarized in Table 1.

All Abs binding the S1 subunit but not the RBD region fell into bin C, while the RBD binders were distributed across the remaining five bins (1 to 5). Bin C- and bin 1-associated Abs did not block binding to ACE2. Bin 2 co-located with the imdevimab epitope, and Abs within bin 2 kinetically perturbed the binding of ACE2, which was confirmed in a waterfall competition assay to be consistent with 23-H7 and ACE2 targeting closely adjacent but non-overlapping epitopes, resulting in a partial blockade. On the other hand, bins 3 and 4 fully blocked ACE2. Bin 3 Abs co-located with casirivimab and etesevimab epitopes, but additionally blocked bin 2. Bin 4, like bin 3, co-located with casirivimab and etesevimab epitopes, while bin 5 co-located with CR3022, VHH-72, and SB68 epitopes. Bin 4 and bin 5 Abs blocked binding to ACE2, but not one another. The clones that fell into bin C bound to the S-protein trimer and S1 but did not bind to the NTD, RBD, or S2. Bin S2 corresponded to subunit 2. Bins C and S2 did not co-locate with any of the benchmark Abs.

**Figure 5:**
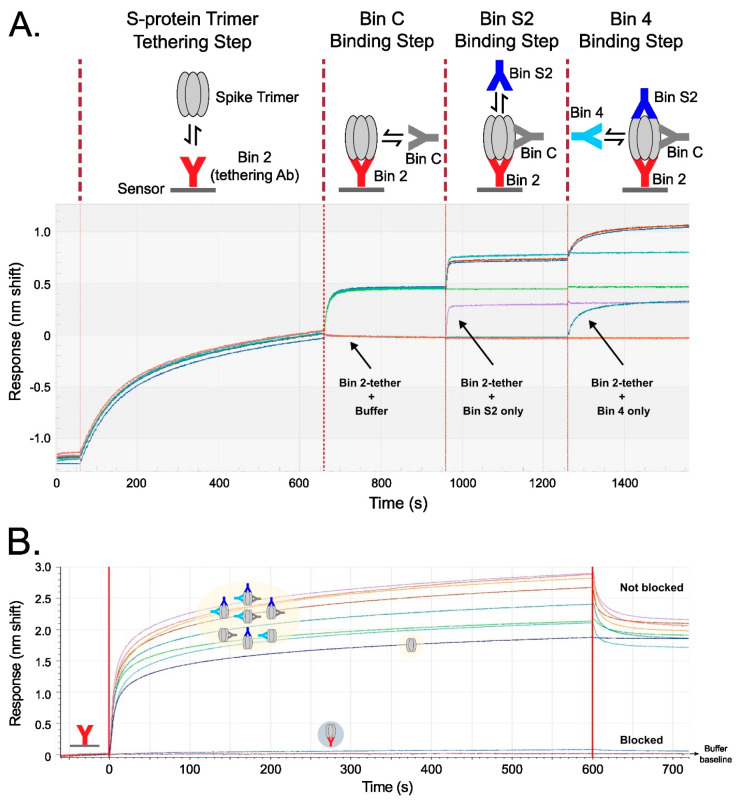
Binning results verifying the simultaneous saturation of spike protein trimer with up to four Abs targeting distinct non-overlapping epitopes in two complementary assay formats. Each TATX-03 Ab was immobilized and tested identically, but examples are given with Ab 23-H7 (bin 2) immobilized to the sensor surface. (**A**) Tandem combination experimental scheme and sensorgrams showing that tethered S-protein allows for the stepwise association of Abs from three other non-overlapping bins represented by 22-E7 (bin C), 2-A6 (bin S2), and 21-F2 (bin 4). (**B**) In a premix format, spike protein was mixed with saturating concentrations of up to three Abs from non-overlapping epitope bins (as indicated in the yellow highlighting). All TATX-03 combinations were tested. As an example, here premixes are presented to a 23-H7-coated sensor, with spike protein alone as control. The colors of the drawn Abs in the light-yellow box correspond to those in the model depicted in Figure 3: 23-H7 (bin 2, red), 22-D9 (bin 4, turquoise), 22-E7 (bin C, grey), and 2-A6 (bin S2, blue).

The six lead Abs of TATX-03 were further investigated in higher-order binning experiments. Two complementary biosensor assay formats, a tandem combination and a premix assay, were used (Figure 5). The sensorgram from the tandem combination showed a clear stepwise addition of four Abs binding simultaneously to the recombinant fully assembled spike trimer. The premix format provided saturating concentrations of Abs binding to the trimer, confirming a true non-blocked response, as opposed to binding to empty epitopes. In contrast, when the premix contained the same Ab as that fixed to the sensor, the spike trimer was blocked from binding the Ab tether, seen as a band running even with the buffer baseline. Together, the two formats support the prediction of our pairwise analyses: when selected from across bins, multiple Abs can decorate the spike without steric hinderance. In the premix run shown in Figure 5B, the lowest curve in the overlay sensorgram corresponds to non-decorated S-protein trimer binding to immobilized 23-H7, whereas the trimer premixed with three or two Abs correspond to the highest bands, and the complexes with one Ab are in between. The avidity of the non-premixed S-protein trimer–antibody complex prevented dissociation from the immobilized 23-H7 antibody (the blue curve in Figure 5B).

Viable immunotherapies not only require adequate target binding reactivity but also must retain certain biophysical properties which enable the stability of the final drug product and efficient manufacturing. The clinical developability of the six lead Abs, such as their (thermal) stability and monomericity values, was explored by size-exclusion (SEC), cross-interaction (CIC), and standup monolayer (SMAC) chromatography, capillary electrophoresis (CE), and a PEG4000-induced turbidity analysis.

The results were compared to benchmarks with good and poor biophysical properties, including bococizumab, CNTO607, NISTmAb, and trastuzumab (see Figure S1 for an example of such a data set). The capillary electrophoresis of 21-F2 and 22-D9 under reducing conditions showed two prominent peaks for the heavy chain of each clone instead of a single signal, suggesting partial glycosylation (Figure S1). Homology modeling (Schrödinger BioLuminate, Schrödinger, Mannheim, Germany, version 2021-01 template PDB ID:5WL2) of the Fv portion and a subsequent structure-based analysis for reactive residues identified a solvent-exposed, potential N-glycosylation site in the Framework 1 region of Ab 22-D9 (N20) and one in the Framework 3 region of Ab 21-F2 (N92). Single-point mutations destroying the identified N-glycosylation tripeptide motif were introduced to remove the original microheterogeneity. A repeated CE analysis confirmed the heavy chain homogeneity of the optimized 21-F2(N92Q) and 22-D9(N20K) Abs. Compared to their partly N-glycosylated parent molecules, their ELISA binding profiles and pseudovirus neutralization capacities (see below) were virtually identical, suggesting that the modification did not affect the target reactivity of the mutated variant Abs.

Analyses of the six Abs by CIC showed retention times within an 8% range of the well-behaved NISTmAb reference, except for Ab 2-A6, which showed prolonged retention, suggesting a lower solubility by non-specific binding to immunoglobulins [46]. For the Fv model of Ab 22-D9, the calculated AggScore, which is predictive for aggregation-prone protein regions [47], was higher than that of the clinical reference, trastuzumab. Adapted Ab 22-D9(N20K), but not clone 2-A6, showed an onset of turbidity at approximately 6% (*m*/*v*) PEG4000, similar to the aggregation-prone reference, CNTO607 [30]. Despite these findings, an SEC-HPLC analysis of gel-filtrated Abs 2-A6 and 22-D9 stored in PBS buffer for at least one month at 4 °C showed >99% monomericity. Nevertheless, the less favorable physicochemical properties of 22-D9, its unfavorable retention time on SMAC-HPLC, and poor solubility hindered some of the experiments, as discussed below.

### 3.2. In Vitro and In Vivo Neutralization Effects of TATX-03 Abs

Individually and in combinations of two, three, four, and five, lead Abs were tested in cell-based pseudovirus and authentic (viable) virus neutralization assays. To plan these experiments pragmatically, functionally complementary pairs of Abs were first identified (Figure 3) and used to anchor higher-order Ab combinations of TATX-03a and TATX-03b. Although 22-D9 and 21-F2 were both assigned to bin 4, their epitopes were apparently not identical as shown in the pairwise competition analysis and by their distinguishable reactivity with some of the viral strains (see below). Even though these two Abs competed for an overlapping epitope, a five-Ab combination (TATX-03c) containing both of these clones was rationalized to take advantage of their subtle epitope differences that might afford compensatory advantages.

**Figure 6:**
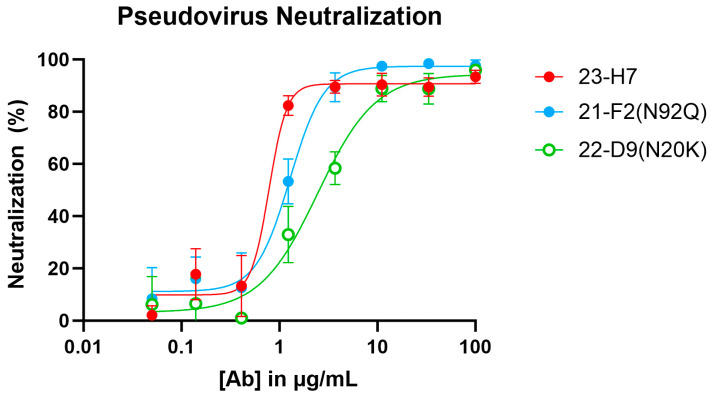
Pseudovirus (Wuhan-Hu1 isolate S-protein sequence) neutralization by individual clones in bins 2, 4, C, and S2. [Ab]: antibody concentration.

**Figure 7:**
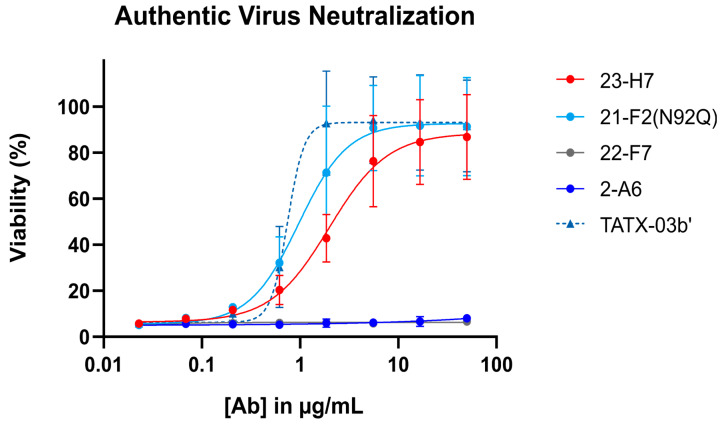
Authentic (viable) virus (EU-1 strain) neutralization by single Abs (indicated) and the four-Ab combination TATX-03b′. Top tested concentration was 50 μg/mL for all Abs and for TATX-03b. Colors of curves and points correspond to those introduced in Figure 3: 23-H7 (bin 2, red), 21-F2(N92Q) (bin 4, turquoise), 22-F7 (bin C, grey), and 2-A6 (bin S2, blue). [Ab]: antibody concentration.

When tested alone in the pseudovirus neutralization assay, the neutralizing Abs showed a concentration-dependent, but variable activity (Figure 6). As expected, no clear neutralization was obtained with 2-A6 (bin S2) and 22-F7 (bin C), whereas 23-H7 (bin 2), 22-D9 (bin 4), and 21-F2 (bin 4) resulted in 100% neutralization at the highest tested concentration. The authentic virus neutralization assay confirmed these results with only 23-H7 and 21-F2(N92Q) showing potent neutralization (Figure 7) and no observed activity from the non-RBD blockers 22-F7 and 2-A6. In this experiment, neither Ab 22-D9 nor its optimized ‘N20K variant’ were tested for reasons explained above.

**Figure 8:**
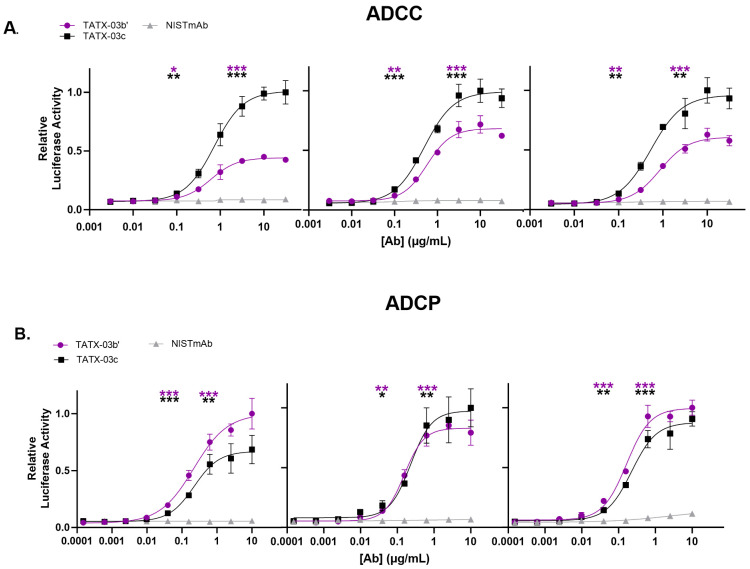
Cellular FcγR activation by Ab combination TATX-03b′ and -03c, or IgG_1_ isotype control Ab in the presence of SARS-CoV-2 target cells and (**A**) Jurkat cytotoxicity reporter cells (ADCC) or (**B**) THP-1 phagocytosis reporter cells (ADCP). Cellular FcγR activation was measured through luciferase reporter activity. Inter-assay results were normalized. In each independent analysis run (panel), the highest averaged luciferase activity was set to 1. The significance of the difference in FcγR activation between TATX-03b′(purple) or TATX-03c (black) and NISTmAb (grey) at the lower and upper inflection points is indicated with asterisks: * *p* ≤ 0.05; ** *p* ≤ 0.01; *** *p* ≤ 0.001. [Ab]: antibody concentration.

In addition, the ability of the Abs, either individually or in combination, to activate secondary effector functions was investigated with antibody-dependent cellular cytotoxicity (ADCC) and phagocytosis (ADCP) reporter assays (Figure 8). In these tests, reporter cells were not non-specifically activated when incubated with Abs in the absence of S-protein-bearing CHO-K1 cells. The control Ab, NISTmAb, did not activate reporter cells expressing FcγRs in the presence of S-protein-positive cells either. The combinations TATX-03b′ (23-H7, 21-F2(N92Q), 22-F7, 2-A6) and TATX-03c′ (23-H7, 21-F2(N92Q), 22-D9(N20K), 22-F7, 2-A6) induced significant FcγRIII receptor-dependent signaling of Jurkat cytotoxicity reporter cells (ADCC). In the ADCP model, TATX-03b′ and TATX-03c were able to significantly activate THP-1 reporter cells. Here, 22-D9(N20K) was soluble and functional by adapting the TATX-03c′ buffer to 20 mM histidine at a pH of 5.3 containing 8.8% trehalose and 0.025% Tween-80.

**Figure 9:**
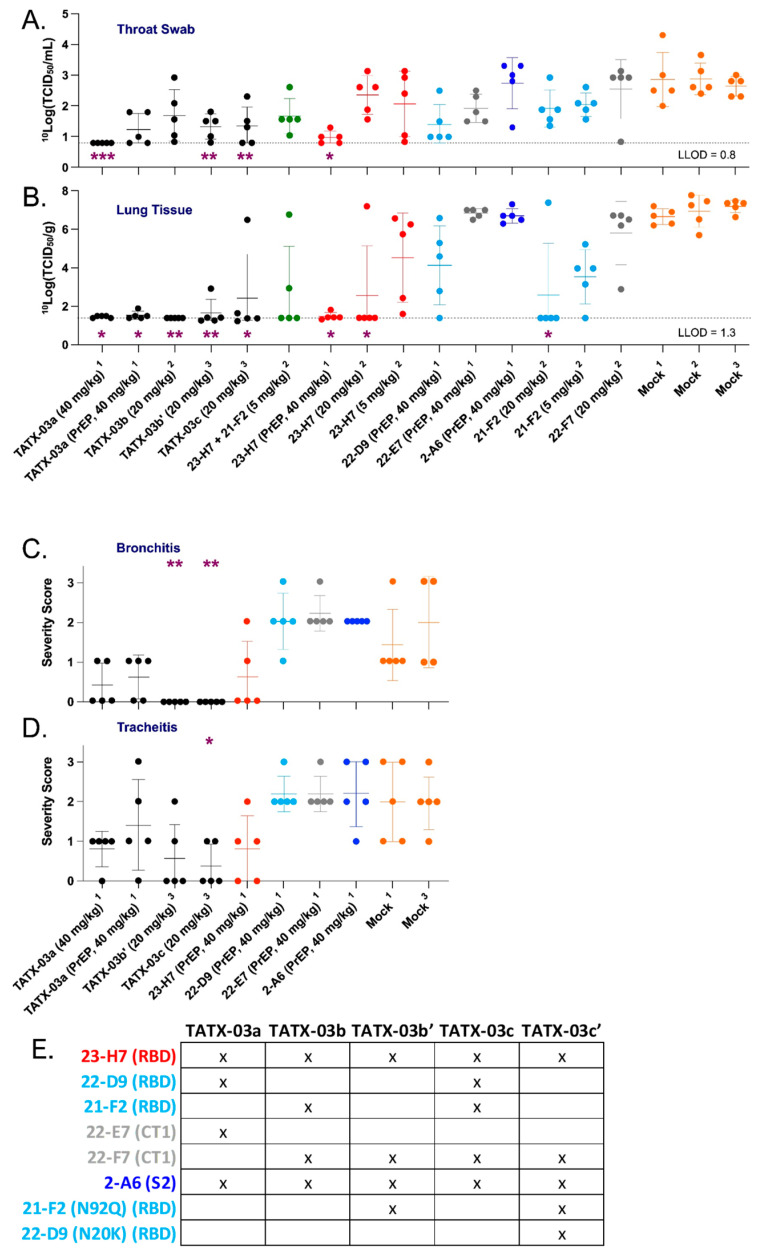
SARS-CoV-2-infection-challenged Syrian golden hamsters. Viable virus loads were determined in (**A**) throat swabs and (**B**) lung tissue. In addition, the main airway inflammation parameters (**C**) bronchitis and (**D**) tracheitis were also determined, reflecting the in vivo efficacy of combinations and individual Abs in blends TATX-03a, TATX-03b(′), and TATX-03c(all black dots) at indicated doses, and compared to those of mock (amber dots) treated hamsters. Administration prior to exposure to the virus is indicated by PrEP (pre-challenge prophylactic). The results of three independent animal experiments are compiled; the results per experiment are indicated by the superscripts ‘1’, ‘2’, or ‘3’. The colors introduced in Figure 3 are used to indicate the results for treatment with clone 23-H7 (bin 2, red), 21-F2 and 22-D9 (bin 4, turquoise), 22-E7 and 22-F7 (bin C, grey), and 2-A6 (bin S2, blue). The level of significance of the results compared to a mock treatment is shown by one or more purple asterisks at the bottom (Band C) or top (Dand E) of the Figures: * *p* < 0.05, ** *p* < 0.01, and *** *p* < 0.001. See Section 2 for more details. (**E**) Summary table depicting the composition of each of the different antibody cocktails.

The in vivo efficacy of TATX-03a, TATX-03b, TATX-03b′, TATX-03c Ab combinations (Figure 9E), and their constituent Abs was tested in a validated Syrian hamster model of acute SARS-CoV-2 infection in three independent animal studies [48,49]. Experimental Ab-containing solutions (that were gel-filtrated just prior to their use) and PBS mock solutions were administered as a single intraperitoneal injection either 24 h pre- or 4 h post-challenge with a single intranasal dose of 10^2.0^ TCID_50_ infectious SARS-CoV-2 as a pre-exposure prophylaxis (PrEP) or post-exposure treatment (PET), respectively (Figure 1). Successful infection was confirmed in all of the animals by real-time PCR (RT-PCR) testing for the presence of the virus on day 1 in the throat swab samples. All animals survived to the endpoint on day 4 post-challenge, but they all lost body weight with no significant difference between any cohort on any day of the study.

The pre-exposure TATX-03a treatment with the 40 mg Ab combination per kg bodyweight (bw) (equivalent to 10 mg/kg bw/Ab) showed clear replication-competent virus titer reductions in the animals’ throat swabs on day 3 compared to the mock-treated group. Titers close to or below the lower limit of detection (LLOD) were determined in all of the sampled PET-treated animals (5/5) dosed at the same concentration (Figure 9A). Treatment with a lower post-challenge dose of TATX-03a, TATX-03b′, or TATX-03c (20 mg/kg bw total Ab) revealed viable viruses in most of the day 3 throat samples in each cohort.

At the day 4 endpoint, coinciding with the end of the acute phase of the infection in the hamster model, whole lung tissue, the key organ in the early etiology of COVID-19, was collected from the PrEP and PET TATX-03a-treated cohorts (40 mg/kg bw). An analysis of the samples demonstrated a statistically significant reduction in viable virus titers compared to those of the mock-treated group (Figure 9B), with 100% (5/5) and 80% (4/5) of the PET and PrEP-treated animals, respectively, showing titers at or below the LLOD. At half this dose, 20 mg/kg bw total Ab, replication-competent virus was either undetectable (TATX-03b) or otherwise significantly reduced (TATX-03b′ or TATX-03c) compared to the mock-treated cohort following PET treatment. Compared to the Ab combinations, only high-dose monotherapy with 23-H7 (40 or 20 mg/kg bw) or 21-F2 (20 mg/kg bw) led to similar efficacies with viable virus titers at or close to the LLOD in lung tissue (Figure 9B).

When used at a concentration identical to that in the TATX-03b blend, namely 5 mg/kg bw, 23-H7 or 21-F2 monotherapy resulted in a much lower titer reduction in lung tissue. Reduction of viral load at 40 mg/kg bw, was limited and not conclusive for 22-D9 and negligible for 22-E7 and 2-A6 (Figure 9B). The 23-H7 and 21-F2 dual Ab combination dosed at 5 mg/kg bw total Ab (2.5 mg/kg bw/Ab) was more potent than either individual Ab administered at 5 mg/kg bw and resulted in undetectable levels in 60% (3/5) of the animals. At this dose, monotherapy resulted in levels at or near the LLOD in only 20% (1/5) of the animals. These findings indicate that the efficacy of the four and two Ab mixtures cannot solely be attributed to the presence of 23-H7 or 21-F2 alone.

**Figure 10:**
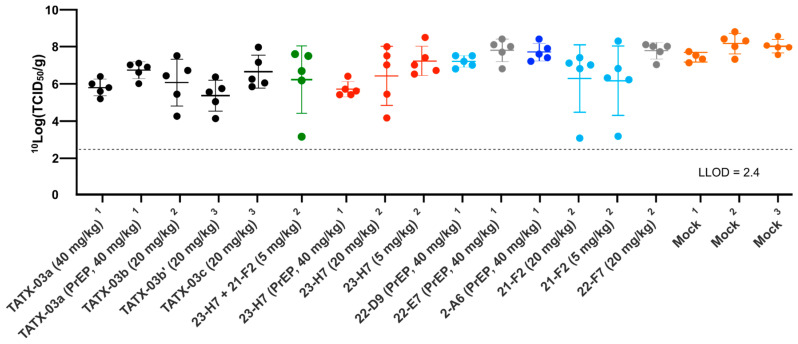
Replication-competent viral titer in nasal turbinate collected from SARS-CoV-2-challenged Syrian golden hamsters at day 4. The results of three independent animal experiments are compiled. Each independent experiment is indicated by superscripts ‘1’, ‘2’, or ‘3’. The colors introduced in Figure 3 are used to indicate the results for treatment with clone 23-H7 (bin 2, red), 21-F2 (bin 4, turquoise), 22-F7 (bin C, grey), and 2-A6 (bin S2, blue), a combination of 23-H7 and 21-F2 (green) and mock treatment (amber).

In line with the low levels of viable virus in this tissue, compared to the mock-treated cohort, lung damage was reduced by the TATX-03 treatment as bronchitis and tracheitis did not occur or were limited (Figure 9C,D). Despite the reduced viral burden in the lower respiratory tract by the treatment, all animals harbored detectable viable viral titers in the nasal turbinate at the endpoint (Figure 10). This observation is in line with previous reports of discordant reductions in viral load between lung and nasal turbinate in this infection model [50], which is anticipated to be due to prominent local infections following intranasal inoculation with a significant bolus of the virus.

**Figure 11:**
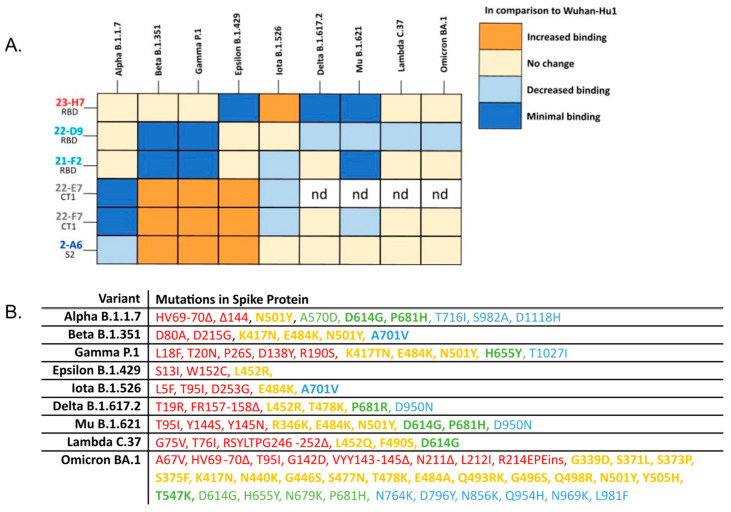
(**A**) Heatmap summarizing the complementary vulnerabilities of individual TATX-03 components towards cell-associated S-protein trimer variants. Cells transfected with an empty expression vector were used as negative control and did not show reactivity. Relative reactivity compared to the Wuhan-Hu1 variant is indicated by different colors. nd: not determined. (**B**) Detailed amino acid mutations included in the variants screened in the cell-based assay. Bolded terms are key mutations as indicated by the ECDC [51]. Colors of each mutation correspond the to the region of the spike protein that is affected as in Figure 4: NTD amino acids 14-306 (red); RBD, amino acids 331-528 (yellow); CT1 of S1 subunit amino acids 529-685 (green); and S2 amino acids 686+ (blue) [52].

The added value of Ab 22-D9 in TATX-03a and TATX-03c compared to TATX-03b(′) was limited or undetectable. Moreover, the reactivity of this clone towards the VoCs, Delta, Mu, Lambda, and Omicron, was inferior (Figure 11). Therefore, the continuation of the development of TATX-03b′ over TATX-03a was obvious, as well as over TATX-03c, as the four-Ab mixture was comparably effective in reducing the monitored COVID-19-related disease markers, including viral load reductions in throat swabs and lung tissue, and inflammation scores, despite the extra Ab (Figure 9A–D).

### 3.3. Four-Ab Combination Overcomes Escape by Variants of Concern (VoCs)

**Figure 12:**
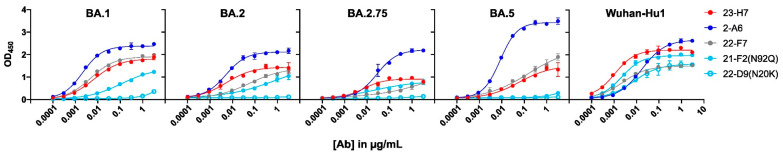
ELISA-based reactivity screening of individual components of TATX-03 to plate-immobilized spike trimer bearing the hallmark mutations of indicated Omicron sublineages or Wuhan-Hu1 trimer as indicated. Colors correspond to those appointed to the bins defined in Figure 3. [Ab]: antibody concentration.

In addition to immune effects, the binding resilience of an antibody-based therapeutic towards an evolving virus is crucial for a durable product. To determine whether the components of the TATX-03 combinations were reactive to SARS-CoV-2 VoCs, individual Abs were screened against a panel of S-protein trimers harboring mutations from B.1.1.7 (Alpha), B.1.351 (Beta), B.1.617.2 (Delta), P.1 (Gamma), B.1.621 (Mu), B.1.429 (Epsilon), and B.1.526 (Iota); Omicron BA.1, BA.2, BA.2.75, and BA.4/BA.5; and the A (Wuhan-Hu1) and B (D614G) parental lineages as reference. The S-proteins of Omicron variants BA.4 and BA.5 are identical and therefore referred to as BA.4/BA.5 [53]. The binding to the cell-associated (Figure 11) and plate-adsorbed (ELISA) recombinant S-protein suggested an epitope bin-dependent susceptibility to viral variants. The results from both reactivity assays were predominantly in line with each other for all tested VoC mutations. In the ELISAs, a weakened binding of the bin 2, 4, and C Abs with Omicron BA.1, BA.2, BA.2.75, and BA.4/BA.5 was observed (Figure 12).

**Figure 13:**
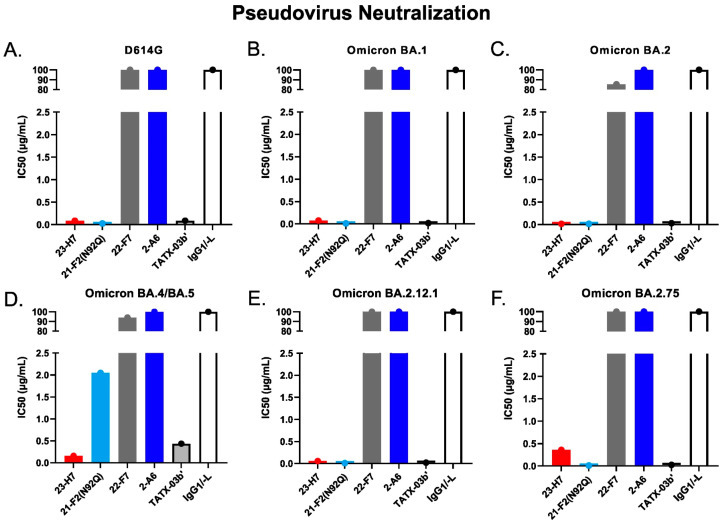
SARS-CoV-2 pseudovirus neutralization by individual Abs and TATX-03b′ combination as per IC_50_. Probed virus variants were (**A**) D614G, (**B**) Omicron BA.1, (**C**) Omicron BA.2, (**D**) Omicron BA.4/BA.5, (**E**) Omicron BA.2.12.1, and (**F**) Omicron BA.2.75. Human IgG_1_/-L was used as negative isotype control. The IC_50_ values are given in µg/mL. The colors introduced in Figure 3 are used to indicate the results for clone 23-H7 (bin 2, red), 21-F2(N92Q) (bin 4, turquoise), 22-F7 (bin C, grey), and 2-A6 (bin S2, blue).

**Figure 14:**
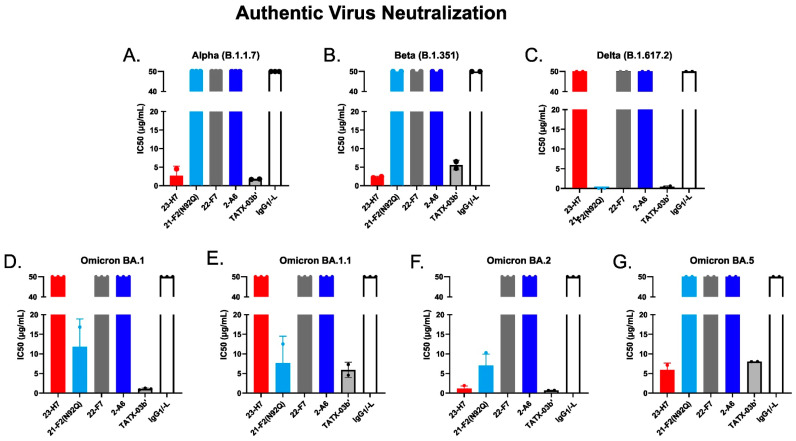
Neutralization of 50% of authentic SARS-CoV-2 virus variants by individual Abs, TATX-03b′ Ab combination, and isotype control (human IgG_1_/-L). Probed virus variants were (**A**) Alpha, (**B**) Beta, (**C**) Delta, (**D**) Omicron BA.1, (**E**) Omicron BA.1.1, (**F**) Omicron BA.2, (**G**) Omicron BA.5. The IC_50_ values are given in µg/mL. The colors introduced in Figure 3 are used to indicate the results for clone 23-H7 (bin 2, red), 21-F2(N92Q) (bin 4, turquoise), 22-F7 (bin C, grey), and 2-A6 (bin S2, blue).

Despite its coinciding patterns, differences were observed for RBD binder Ab 21-F2(N92Q), which underperforms towards the Omicron subvariants in the ELISA but retains its binding activity in the cell-based assay. Accordingly, 21-F2(N92Q) inhibited cell infection of all pseudotyped VSV particles tested in the in vitro neutralization assays (Figure 13, Table 2). Similarly, the blocking of cell entry by 21-F2(N92Q) was effective for all Omicron subvariants except for BA.4/BA.5 in the authentic virus assay (Figure 14, Table 2).

Non-RBD Abs 22-E7 and 22-F7 (both bin C) showed variable changes in binding to all tested cell-associated S-protein trimer variants compared to Wuhan-Hu1with no binding to B.1.1.7 (Alpha). The marginally different blocking profiles of bin-4 Abs, 22-D9, and 21-F2, observed in their discovery phase (see above), were reflected in their reactivity to cell-exposed S-protein trimers representing Delta, Iota, Mu, Lambda, and Omicron (Figure 11). Bin S2 (2-A6) showed uniform binding to all tested mutant trimer constructs, including those of Omicron subvariants (Figure 12), but failed to neutralize any of the tested pseudovirus or authentic virus variants in the respective in vitro assay.

The functional consequences of the key antigen mutations were not observed for the TATX-03 antibody combinations. They retained their neutralization potency against the pseudotyped viruses adapted to the Alpha, Beta, Delta, and Omicron BA.1, BA.2, BA.2.12.1, BA.2.75, and BA.4/BA.5 variants. The maintained neutralization of TATX-03b′ was confirmed by authentic virus neutralization screenings using a panel of SARS-CoV-2 virus variants (Figure 14, Table 2)

## 4. Discussion

The applied antiviral activity screening funnel reduced the 700 initially recovered clones from two SARS-CoV-2-naïve phage libraries down to 60 unique-sequence binders for further analysis. Unlike many other studies developing mono- or dual-Ab immunotherapies [54], the binding reactivity of the Ab candidates was not optimized by a sequence maturation process. Instead, we hypothesized that a broad epitope footprint by simultaneously binding multiple anti-SARS-CoV-2 antibodies is not only an encouraging approach for a durable resilience against an immune-evading virus but also a strategy to unlock more than one immune effect or mechanism in an additive or synergistic way. Neutralization breadth may provide a better guarantee for VoC-agnostic potency than one or two high-affinity Abs [8]. Moreover, the mobilization of concerted secondary effects due to a high level of Ab decoration has been shown to outperform the neutralizing effects of a single high-affinity binder [55]. A similar line of therapeutic defense, i.e., mutant resilience and joint functionalities, was recommended for the use of a combinatorial therapy of antivirals [56]. In contrast to many studies [57], the Ab combination in this study aims at a more diverse epitope coverage than that of the RBD region alone. A combination of a blocking and non-blocking binder is pursued in bispecific VH/Fab antibodies demonstrating a higher neutralization potency compared to that of their monospecific bivalent counterparts [58]. In a similar way, IgG-like bispecific antibodies (tandem scFv-Fc) demonstrated an improved virus neutralization potency through synergistic activity [54]. Of all other non-RBD regions considered for our multi-epitope binders, the NTD was purposely ignored, as it is a low-quality epitope due to its high mutability and high sensitivity for conformational changes [57], and, consequently, anti-NTD Abs may lose their fitness rapidly.

While aiming for a high-order combination of multiple full-length human Abs with intact Fc functionality and between which only the variable domains differ, it is a promising observation that concurrently administered antibodies show generally no or very little coincidental toxicity [59]. Whereas most studies define cocktails and combinations as a blend of only two Ab components, this study aimed at oligoclonal mixtures of at least four Abs.

Whereas one can speculate on the intricacy of clinical and product development of combination therapies compared to monotherapies, such facets may be negligible in the light of boosted efficacy, allowing for lower dosing and increased market longevity due to improved resilience against new virus mutants.

The definition of the six bins represented an over-simplification of a more nuanced epitope landscape. However, the defined bins were adequate to compose a functional and resilient TATX-03 Ab combination. The simultaneous binding of the constituents of the combination to the S-protein was demonstrated, and further refinement or confirmation of hypothesized bin structures by, e.g., cryogenic electronic microscopy, was deemed unnecessary.

In the composed TATX-03 combinations, the clones 21-F2 and 22-D9 (both bin 4) and 23-H7 (bin 2) target similar S-protein regions as casirivimab (REGN10933) and imdevimab (REGN10987) in REGN-CoV2, which, when bound to these regions, disrupt the interaction between the RBD and ACE2 of the host. In the epitope landscape presented by Hastie et al., bin 2 co-locates with RBD-5 on the S-protein, which shows a high level of sequence conservation [57]. In this map, bin 4 correlates to RBD-2, which tends to be more vulnerable for the loss of neutralization potency by Abs binding this region [57]. These region-specific traits reflect the epitope bin-dependent susceptibility to viral variants, showing that bin 4 clones 22-D9 and 21-F2 are apparently more affected by the mutations in the tested variants than any other clone (Figure 11 and Figure 12), whereas bin 2 clone 23-H7 binds, for example, all probed Omicron sublineages (Figure 12).

Interestingly, in contrast to bin 2 benchmark antibodies, such as imdevimab, RBD blocker 23-H7 (bin 2) retained binding towards BA.4/BA.5 (Figure 12) despite the presence of the L452R mutation in bin 2. Apparently, bin assignments contain nuances within them despite the two Abs cross-blocking one another, and they can individually be affected by different mutations. This was also observed for the epitope-competing bin 4 Abs, 21-F2 and 22-D9, which showed subtle epitope differences.

In the case of Ab 23-H7, an apparent difference in functional inhibition is observed in the pseudovirus and the authentic virus neutralization assays (see Figure 13 and Figure 14). This is anticipated to result from the application of different ACE2-expressing cell lines in these assays. Compared to several other epithelial cell types, VeroE6 cells express ACE2 the strongest [60]. The MDA-MB-231 cells in the authentic virus neutralization assay are known to overexpress human ACE2 [61]. The abundancy of ACE2 receptors on these cell-lines may have biased the results for the RBD binder 23-H7, which was found to give only a partial blockade of the binding to ACE2.

A functional triage exclusively based on the RBD-ACE2 blockade would eliminate clones not showing this interference. In this study, two epitope bins (C and S2) outside the RBD of the S-protein were included in TATX-03 to engage other MoAs. Among the selected non-RBD-blockers, Ab 2-A6 binds to the S2 region, which is considered a highly conserved domain and therefore a promising target for a sustainable therapeutic [62,63,64,65]. However, Abs recognizing this region of the spike trimer are rarely reported.

The TATX-03 treatments provided good to complete protection to virus-exposed hamsters with minimal changes in gross pathology, limited lung damage, and importantly, significantly reduced viable virus titers in the respiratory tract compared to the mock-treated groups. The constituent Abs binding of the RBD of the spike protein, 23-H7, 21-F2, and 22-D9, demonstrated a partial to a significant reduction in viable virus counts and a lower level of lung damage. However, the findings indicate that the efficacy cannot solely be attributed to the presence of the RBD-blockers, 23-H7, 21-F2, or 22-D9, alone. At a concentration identical to those in the TATX-03 mixtures, or at an identical total protein concentration of the dual 23-H7 plus 21-F2 combination, the monotherapies reached a much less pronounced viral titer reduction in, e.g., lung tissue. Here, an obvious conclusion is that when the TATX-03 Abs are combined, they work in concert with their effects at least adding up and possibly acting synergistically.

Besides preventing host entry, FcγR-driven immune effector activities by the fully human Abs in TATX-03 most likely play a strong and durable role in humans. This is supported by the significant signaling of human FcγR-expressing Jurkat and THP-1 reporter cells when brought in contact with TATX-03 Abs and S-protein expressing cells, suggesting that ADCC and ADCP mechanisms, respectively, will be involved in a possible therapy. It is expected that bin S2 binder 2-A6, which activates ADCP (Figure S2), can augment the function of the neutralizing clones from bins 2 and 4 in vivo.

Clusters of multiple antigen-attached Abs bind with a higher affinity to functional Fc receptors and form more stable complexes. In this way, co-bound Abs trigger a more effective viral load reduction than monomeric Ab-antigen complexes [55,66]. Unfortunately, the interspecies differences between hamster and human FcγR orthologs [67] may prevent an optimal fit of the human Fc sequence to the hamster FcγR leading to an underestimation of the neutralization effect in hamster efficacy studies. This difference is probably reflected in the negligible effectiveness of non-RBD-binders 22-F7 and 2-A6 to reduce titers of viable virus in lung tissue (Figure 9B) and thus to protect the lower respiratory tract of the hamsters from damage (Figure 9C,D). Besides the interference of RBD-ACE2 docking and triggering ADCC and ADCP, other reported MoAs are, e.g., the induction of complement-dependent cytotoxicity (CDC), locking the S-protein to acquire its active up conformation, cross-linking S-protein monomers and/or trimers, the degradation of the trimer S-protein structure, and the prevention of the necessary cleavage of the S1/S2 subunit junction [9,68,69,70]. Further investigations are needed to unravel the full functional diversity of the virus-neutralizing MoAs by the simultaneous attachment of the four TATX-03 Abs to the S-protein.

The continued emergence of new viral variants has impacted immunotherapy effectiveness, as well as the immunity acquired through vaccination and infection. The recent XBB Omicron sublineages show the most extensive immune escape observed so far [71]. The Omicron CH.1.1 and CA.3.1 variants are highly resistant to mono- and bivalent mRNA vaccines [4].

Which redundancy and therapeutic qualites are required to cope with current and future VoCs? Even though our selections are guided by their reactivity against the parental Wuhan-Hu1, TATX-03 Ab combinations showed in vitro neutralization potency efficacy against sixteen tested VoCs from Wuhan-Hu1 to Omicron BA.4/BA.5, whereas other immunotherapies showed a much smaller reactivity spectrum [13]. The established broad reactivity of TATX-03 by including at least four combinatorial full-length and complete human Abs targeting multiple distinct and non-overlapping epitopes is strong support for the strategy to design oligoclonal immunotherapies. In addition, current information on the mutability of S-protein regions reveals the importance of targeting non-immunodominant, stable regions with critical functions in the S-protein [8,72]. A combination of two of Abs discovered from B cells harvested from vaccinated convalescents neutralized Omicron variants BA.2.75, BA.2.75.2, BF.7, BQ.1, BQ.1.1, CH.1.1, XBB, and XBB.1 [8]. In TATX-03, clones 2-A6 and, to a lesser extent, 22-E7 bind to regions relatively less prone to mutations, with 2-A6 binding to the S2 subunit involved in the membrane fusion process of the virus with the host cell [64].

Immunotherapies are acknowledged as the best treatment option for (clinically) vulnerable patients, including immunodeficient persons, possibly even when the therapeutic Ab has apparently lost its in vitro efficacy against a new variant [73], although this view of Wu and colleagues has been criticized [74]. This motivates to continue the design of multi-epitope multi-antibody immunotherapies as the best guarantee to provide a safe, tolerable, and stable therapeutic candidate in general and more specifically in disease areas, where prevention of evasion from functional Abs is critical for a full recovery, such as tumorigenesis [75]. 

## Figures and Tables

**Table 1:** The inter-bin relationship deduced from pairwise binning analysis, predicting Abs that can pair together on the spike protein.

Bin	Binding Specificity	Blocks ACE2 Receptor	Blocks Bins	Pairs with Bins	Co-Locates with Benchmark
1	RBD	No	Depending on antibody, members can block 2, 3, 4, C	S2, 5	Imdevimab, Casirivimab, Etesevimab
2	RBD	Partial	1, 3	C, 4, 5, S2	Imdevimab
3	RBD	Yes	2, 4	C, 1, 5, S2	Casirivimab, Etesevimab
4	RBD	Yes	3	C, 1,2,5, S2	Casirivimab, Etesevimab
5	RBD	Yes	none	all	CR3022, VHH-72, SB68
C	S1	No	1	2,3,4,5, S2	none
S2	S2	No	none	all	none

**Table 2:** IC50 values of TATX-03b′ and individual components for each pseudovirus and authentic SARS-CoV-2 virus variant shown in Figure 13 and Figure 14, respectively.

		Antibody					
		23-H7	21-F2 (N92Q)	22-F7	2-A6	TATX-03b’	IgG1/-L
**Pseudovirus** IC50 (µg/mL)	**D614G (VRC8400)**	0.09	0.03	>100	>100	0.09	>100
	**Omicron BA.1**	0.07	0.01	>100	>100	0.02	>100
	**Omicron BA.2**	0.02	0.02	85.39	>100	0.03	>100
	**Omicron BA.4/BA.5**	0.16	2.05	94.08	>100	0.43	>100
	**Omicron BA.2.12.1**	0.05	0.01	>100	>100	0.02	>100
	**Omicron BA.2.75**	0.36	0.01	>100	>100	0.03	>100
**Authentic Virus** IC50 (µg/mL)	**Alpha B.1.1.7**	2.70	ND	NA	NA	1.74	NA
	**Beta B.1.351**	2.32	NA	NA	NA	5.58	NA
	**Delta B.1.617.2**	NA	0.07	NA	NA	0.41	NA
	**Omicron BA.1**	ND	11.82	NA	NA	1.05	NA
	**Omicron BA.1.1**	NA	7.69	NA	NA	5.93	NA
	**Omicron BA.2**	1.15	7.00	NA	NA	0.68	NA
	**Omicron BA.5**	5.90	ND	NA	NA	8.02	NA

Note: TATX-03b′ contains less (1/4) of each individual mAb than was used for determining IC50 of individual mAbs. NA= not applicable.

## Data Availability

The data supporting the conclusions of this article are available on request from the corresponding author.

## Supplementary Materials

The following supporting information can be downloaded at: https://www.mdpi.com/article/10.3390/biomedicines12030642/s1, Figure S1: Example of a developability data set. Figure S2: ADCP induction as measured by cellular FcγR activation in a reporter cell line.

## Abbreviations

Ab	antibody
ACE2	angiotensin converting enzyme 2
ATCC	American Type Culture Collection
AVI	15 amino acid peptide allowing biotinylation
bw	body weight
CDC	complement-dependent cytotoxicity
CIC	cross-interaction chromatography
CHO	Chinese hamster ovary
ECD	extracellular domain
FcγR	Fc gamma receptor
His	histidine
MoA	mechanism of action
NTD	N-terminal domain
PBS	phosphate-buffered saline
PET	post-exposure treatment
PrEP	pre-exposure prophylaxis
RBD	receptor binding domain
RT	room temperature
S1	subunit 1 of the spike glycoprotein
S2	subunit 2 of the spike glycoprotein
scFv	single-chain variable fragment
TMPRSS2	human transmembrane serine protease 2
