# Supplementary material for: Broad Epitope Coverage of Therapeutic Multi-Antibody Combinations Targeting SARS-CoV-2 Boosts In Vivo Protection and Neutralization Potency to Corner an Immune-Evading Virus
Source: Biomedicines. 2024 Mar 13;12(3):642. doi: 10.3390/biomedicines12030642 (PMC10968570; doi:10.3390/biomedicines12030642)
Supplement: Supplementary file 1 [file biomedicines-12-00642-s001.zip › biomedicines-2492542-supplementary.pdf]

Supplementary Materials
Figure S1: Example data for assays described in methods section 2.10-2.13.

1a. *2.10 In Silico Developability Analysis and Manufacturability Optimization*
Example data for the identification of the glycosylation site in the framework of the VH of 22-D9

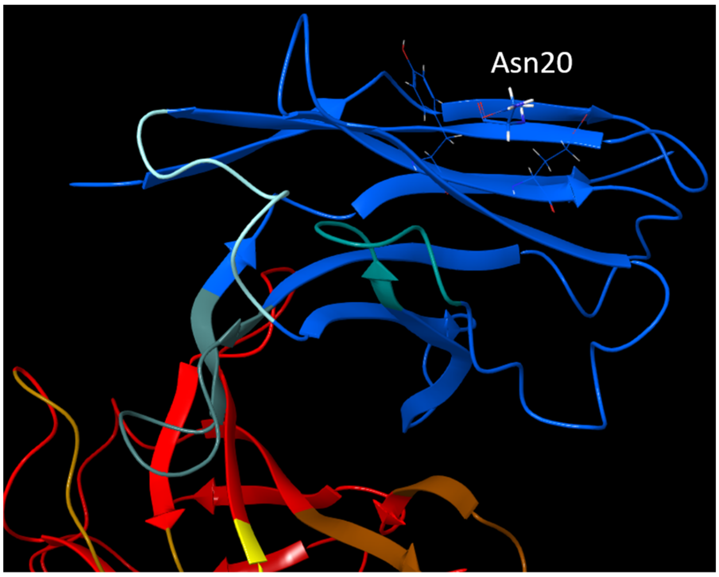

Homology model of 22-D9. The VH domain is presented in blue with the CDR highlighted in different shades of blue. The asparagine at position 20 in framework 1 is solvent exposed and identified as potential glycosylation site.

1b. *2.11 HPLC -Based Analysis*

Example HPLC data for 2 benchmarks and 2 lead mAbs is provided.
2.11.1 Example data SEC-HPLC

NISTmAb

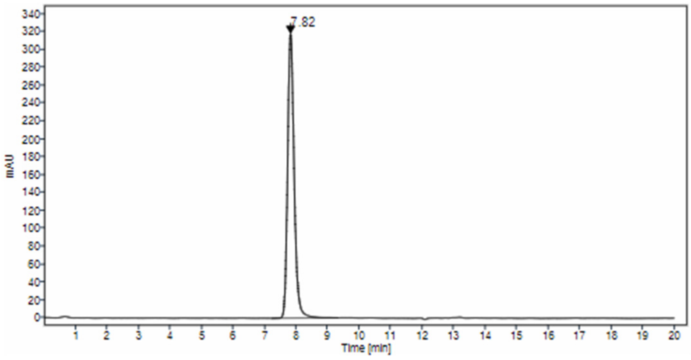

Bococizumab

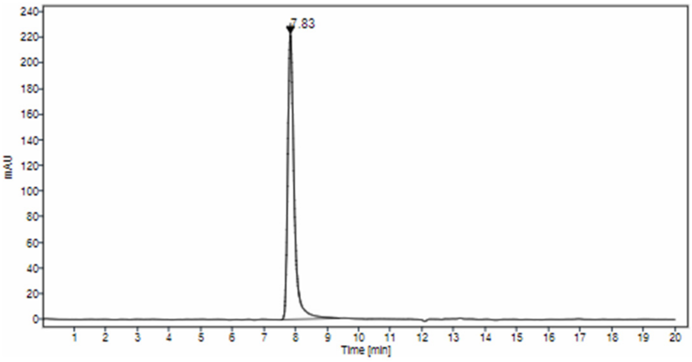

23-H7

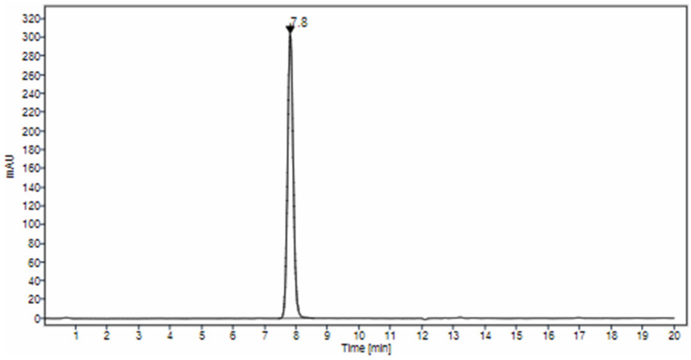

22-D9

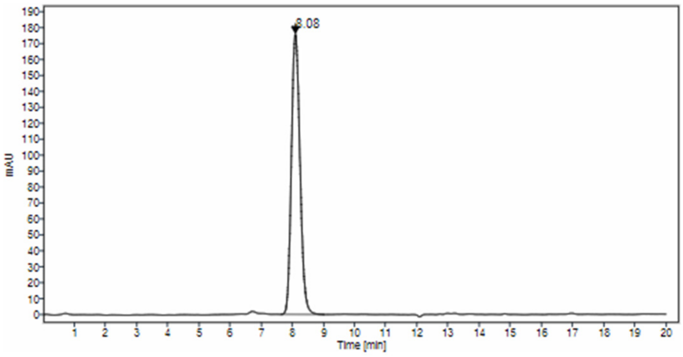

2.11.2 Example data CIC-HPLC

NISTmAb

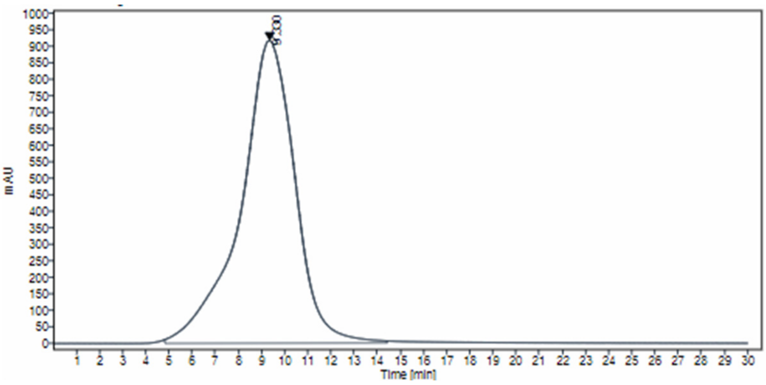

CNTO607

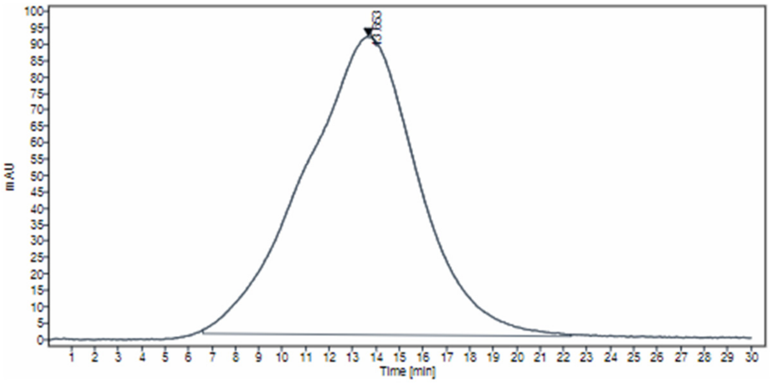

23-H7

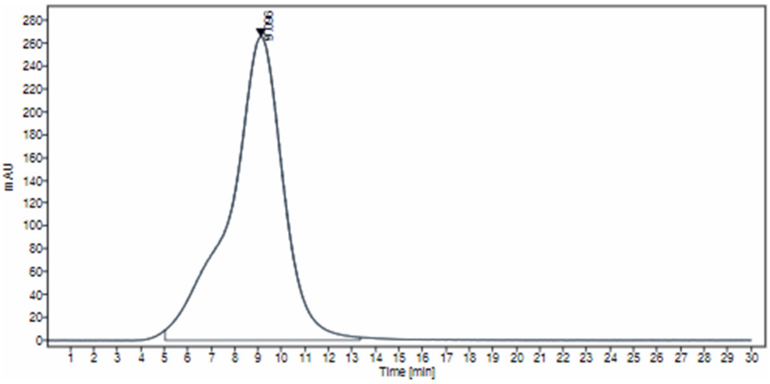

22-D9

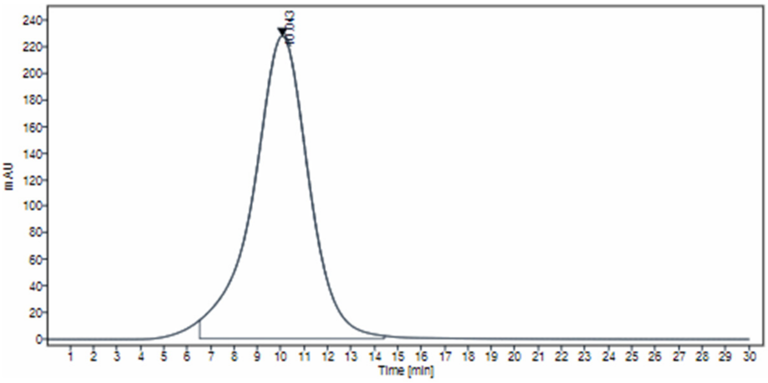

2.11.3 Example data SMAC-HPLC

NISTmAb

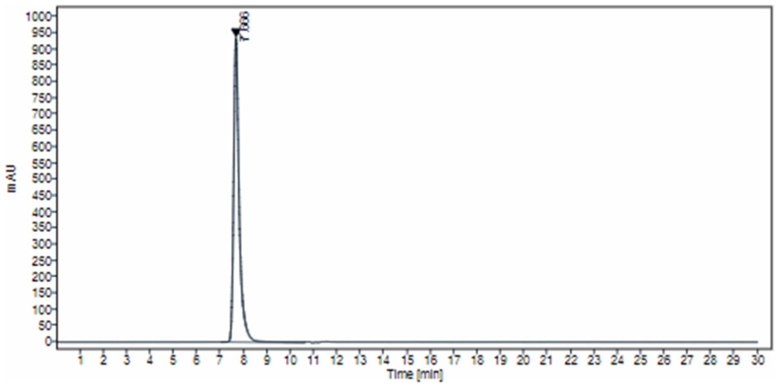

Bococizumab

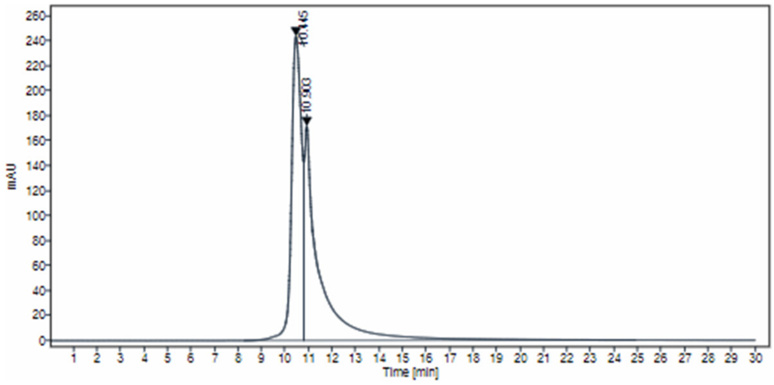

23-H7

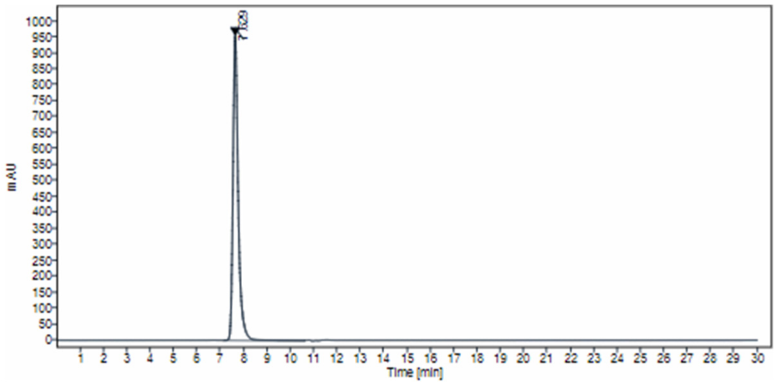

22-D9

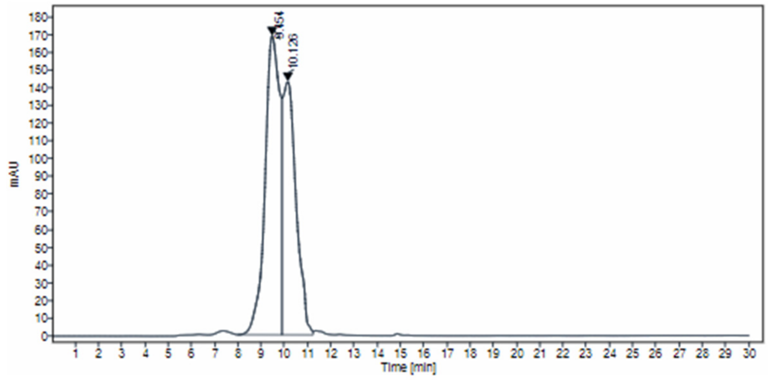

1c. *2.12* *Capillary Electrophroesis (CE)*

Example data CE

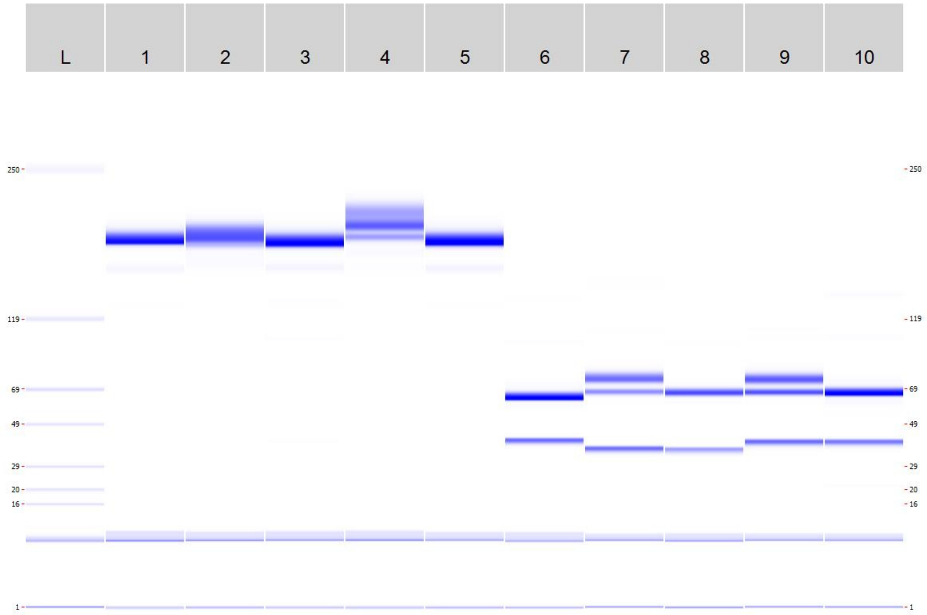

L	Molecular weight marker	Non-reduced
1	23-H7	Non-reduced
2	22-D9-wt	Non-reduced
3	22-D9-N20K	Non-reduced
4	21-F2-wt	Non-reduced
5	21-F2-N92Q	Non-reduced
6	23-H7	Reduced
7	22-D9-wt	Reduced
8	22-D9-N20K	Reduced
9	21-F2-wt	Reduced
10	21-F2-N92Q	Reduced

1d. *2.13 Assessment of Solubility*

Representative example data of the PEG4000 solubility assay

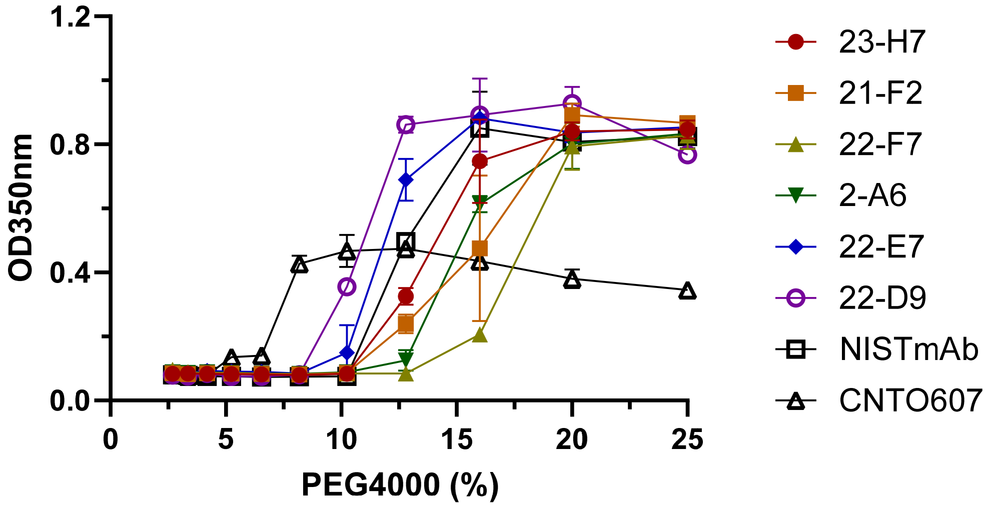

Figure S2. ADCP induction as measured by cellular FcγR activation in a reporter cell line.

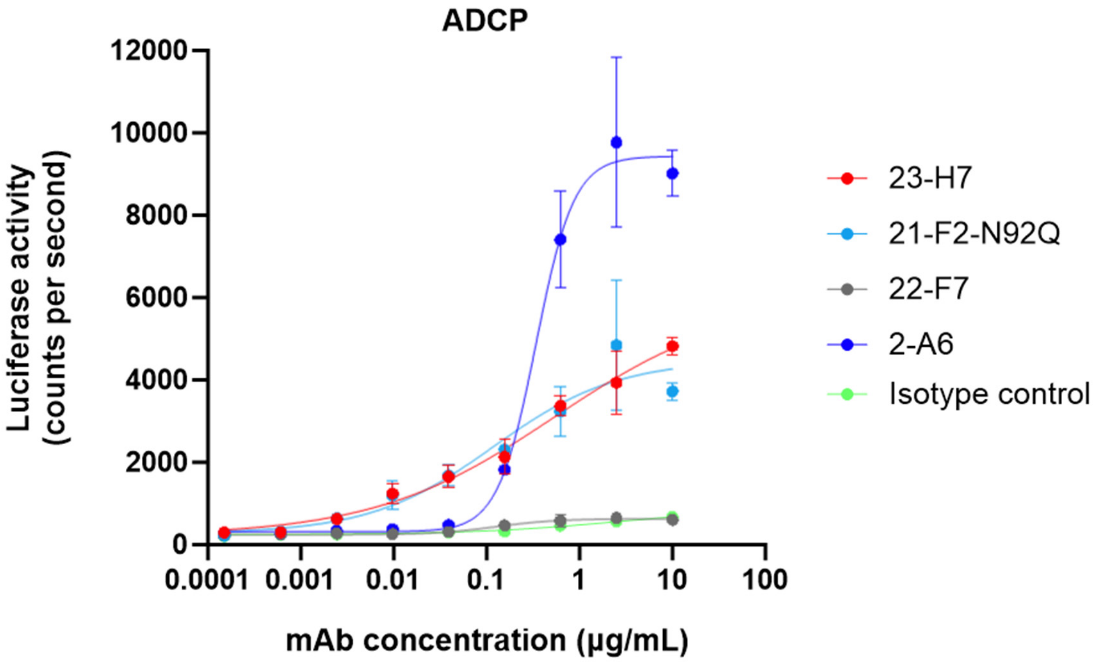

presence of SARS-CoV-2 target cells and THP-1 phagocytosis reporter cells (ADCP). Cellular FcγR activation was measured through luciferase reporter activity. In each independent analysis run, the highest averaged luciferase activity was set to 1.
